# Comprehensive Phylogenetic Reconstruction of Amoebozoa Based on Concatenated Analyses of SSU-rDNA and Actin Genes

**DOI:** 10.1371/journal.pone.0022780

**Published:** 2011-07-28

**Authors:** Daniel J. G. Lahr, Jessica Grant, Truc Nguyen, Jian Hua Lin, Laura A. Katz

**Affiliations:** 1 Graduate Program in Organismic and Evolutionary Biology, University of Massachusetts, Amherst, Massachusetts, United States of America; 2 Department of Biological Sciences, Smith College, Northampton, Massachusetts, United States of America; The Centre for Research and Technology, Hellas, Greece

## Abstract

Evolutionary relationships within Amoebozoa have been the subject of controversy for two reasons: 1) paucity of morphological characters in traditional surveys and 2) haphazard taxonomic sampling in modern molecular reconstructions. These along with other factors have prevented the erection of a definitive system that resolves confidently both higher and lower-level relationships. Additionally, the recent recognition that many protosteloid amoebae are in fact scattered throughout the Amoebozoa suggests that phylogenetic reconstructions have been excluding an extensive and integral group of organisms. Here we provide a comprehensive phylogenetic reconstruction based on 139 taxa using molecular information from both SSU-rDNA and actin genes. We provide molecular data for 13 of those taxa, 12 of which had not been previously characterized. We explored the dataset extensively by generating 18 alternative reconstructions that assess the effect of missing data, long-branched taxa, unstable taxa, fast evolving sites and inclusion of environmental sequences. We compared reconstructions with each other as well as against previously published phylogenies. Our analyses show that many of the morphologically established lower-level relationships (defined here as relationships roughly equivalent to Order level or below) are congruent with molecular data. However, the data are insufficient to corroborate or reject the large majority of proposed higher-level relationships (above the Order-level), with the exception of Tubulinea, Archamoebae and Myxogastrea, which are consistently recovered. Moreover, contrary to previous expectations, the inclusion of available environmental sequences does not significantly improve the Amoebozoa reconstruction. This is probably because key amoebozoan taxa are not easily amplified by environmental sequencing methodology due to high rates of molecular evolution and regular occurrence of large indels and introns. Finally, in an effort to facilitate future sampling of key amoebozoan taxa, we provide a novel methodology for genome amplification and cDNA extraction from single or a few cells, a method that is culture-independent and allows both photodocumentation and extraction of multiple genes from natural samples.

## Introduction

Reconstructing relationships between amoeboid organisms is challenging. Both the perceived and intrinsic paucity of morphological characters when compared to macroscopic taxa, as well as difficulties in establishing homology, made deep inferences nearly impossible for the ∼200 years of studies based on microscopy. As a result, most taxa were lumped into the artificial Sarcodina [1]. However, a number of well-defined morphological groups emerged from morphological information and are rarely disputed [2], including lobose shelled amoebae (the Arcellinida); and the amitochondriate, parasitic amoebae with intra-nuclear mitotic spindles (the Entamoebidae). Major advances were achieved with the use of electronic microscopy techniques, but these generally helped stabilize further the lower-level relationships with additional putative synapomorphies, rather than resolve deep relationships (*eg.* [3], [4]).

With the advent of molecular techniques, amoeboid groups were found to be scattered across at least 30 lineages in the eukaryotic tree of life, with the amoebae producing lobose pseudopodia now included in the Amoebozoa [5]. It was only in the early 2000s that the promise of molecular phylogenetic reconstruction reached the fine-grained relationships within Amoebozoa, with well-sampled analysis of SSU-rDNA and actin genes [6], [7], [8]. The number of available amoebozoan sequences has increased steadily in the last decade, though not exponentially as occurred in other groups. A handful of medically important taxa and model organisms had their complete genomes sequenced or EST data made available (eg. *Dictyostelium discoideum* [9], *Entamoeba histolytica* [10]), but this sampling is still sparse making phylogenomic reconstructions difficult for this diverse group [11]. Currently, there are about 150 diverse strains of Amoebozoa for which the SSU-rDNA has been characterized, followed by the actin gene for a few dozen lineages. These strains basically cover all the traditionally proposed morphological diversity [2], [3], [12].

The last few years provided further stabilization in purported relationships within the Amoebozoa (Figure S1). Two competing classifications emerged almost simultaneously: the higher-level taxonomic system of eukaryotes of Adl et al. [13], and the Amoebozoa system of Cavalier-Smith et al. [14]. Subsequently, both systems were combined using both morphological and molecular evidence in the now standard classification of Smirnov et al. [15]. Numerous additions have been made to the system of Smirnov et al. [15], generally placing *incertae sedis* taxa without much modification into the higher-level proposed relationships (eg. [16], [17], [18], [19], [20], [21], [22]). Subsequent large-scale reconstructions largely corroborated the proposed relationships in the Smirnov et al. [15] system [23], [24], [25], [26]. Notable exceptions to this rule come from analyses of organisms traditionally considered slime molds. The Protostelia, once united by the ability to produce a unicellular fruiting body [27], proved to be scattered in virtually every major branch of the Amoebozoa except for the Tubulinea [28]. In addition, the sorocarpic slime mold *Fonticula alba* was shown to be more closely related to the opisthokont amoebae [29], and *Copromyxa protea* is shown to be in the Tubulinea [30]. The implications and impact of these important insights are yet to be fully appreciated, either: 1) the ability to produce stalked fruiting bodies has emerged multiple times; 2) this ability has emerged once and was either lost or modified many times or; 3) many more lineages of amoebae are able to do so and the differences in the methodological traditions of typological protistology and mycology have failed to take this into account, as suggested by Shadwick et al. [28].

Reconstructing these ancient relationships is an outstanding question difficult to resolve both due to the scattered understanding of the diversity of organisms and the highly heterogeneous rates of molecular evolution within the group [23], [25]. The Amoebozoa may have radiated as early as 1200 Mya [31], with the oldest unambiguous fossil being Arcellinida shells at 750 Mya [32], [33], [34]. Here, we provide a comprehensive reconstruction based on available data, concatenating SSU-rDNA and actin genes for 129 amoebozoan lineages and 10 outgroups. We introduce new molecular data for 13 lineages, 12 of which had not been previously characterized. In order to scrutinize the range of techniques used to reconstruct the Amoebozoa, we explore multiple iterations of taxa and data sampling, aiming to obtain reliable estimates of consistent groups, and to assess critically the support for proposed relationships. We perform comparative analyses using 18 different reconstruction approaches, including differential taxon sampling, removal of fast evolving sites, removal of long-branched and unstable taxa, and inclusion of environmental sequences. We test previously proposed relationships at both lower and higher-levels, and provide a summary of which groups are corroborated given the current molecular and, to a lesser extent, morphological data.

## Results

### 1. General topology

**Figure 1:**
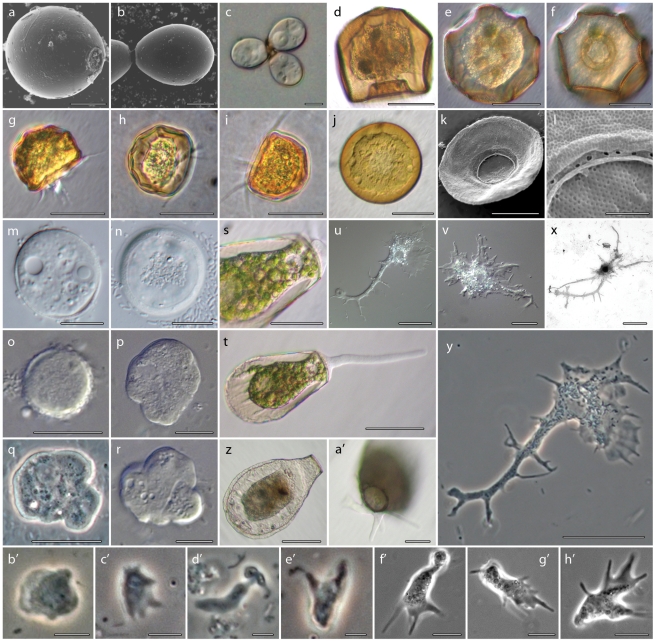
Morphology of the amoeboid lineages isolated for this study. **1a–c.** *Cryptodifflugia operculata*: a) Scanning electron micrograph (SEM) of *C. operculata* in ventral view, showing the distinctive mucous operculum covering the aperture; b) Dorsal view of two *C. operculata* with a cytoplasmic connection, this state is often seen in culture; c) Differential interference contrast images (DIC) of 3 connected *C. operculata* individuals. Scale bars are 5 µm. **1d–f**. Light microscopy images of the *Arcella mitrata* individual that was genome amplified to generate the sequences used in this study: d) lateral view showing the typical polygonal profile; e) top view of the same individual, focal plane at the middle of test height; f) top view of the same individual, focal plane at bottom of test height, showing the characteristic rippled apertural margin. Scale bars are 100 µm. **1g–i**. Hoffman modulation contrast (HMC) images of cultured individuals of *Arcella gibbosa*: g) lateral view showing hemispherical profile and pseudopods; h) another individual showing the shell's ridges and depressions; i) lateral view of a third individual. Scale bars are 60 µm. **1j–l**. *Arcella discoides*: j) HMC image of a cultured individual; k) SEM image showing the thin lateral profile; l) close-up on the apertural margin of individual in k, showing pores surrounding the aperture. Scale bars for j, k are 30 µm, for l 3 µm. **1m–n**. DIC images of cultured *Pyxidicula operculata*: m) focal plane at middle of test height showing the nucleus and one contractile vacuole; n) focal plane at the bottom of a different individual, surrounded by bacteria on which it was feeding. Scale bars are 10 µm. **1o–r**. DIC images of ‘*Govecia fonbrunei*’ ATCC® 50196: o) Encysted individual; p) resting individual, note the hyaline covering visible at the top margin; q) individual shape immediately after excystation; r) initial stages of locomotion. Scale bars are 10 µm. **1s–t**. HMC images of *Hyalosphenia papilio*: s) close up on one of the individuals that was genome amplified to obtain sequences in this study, scale bar 30 µm; t) a more general view of the same individual, scale bar 50 µm. **1u–y**. Images of ‘*Stereomyxa ramosa*’ ATCC® 50982: u,v) Phase contrast images of a cultured individual; x) protargol staining, showing the single nucleus; y) DIC image of a ‘*S. ramosa*’ showing the variety of pseudopods it can produce. Scale bars are 20 µm. **1z–a′**. HIC images of *Nebela carinata*: z) a lateral profile of one of the individuals used to obtain sequences in this study, this image shows the characteristic rim around the margin of the shell; a′) same individual observed in the typical raised shell locomotive position. Scale bars are 20 µm. **1b′–e′**. ‘*Stygamoeba regulata*’ ATCC® 50892: b′) sedentary shape; c′) beginning of movement morphology; d′) start of monopodial movement; e′) polypodial movement. Scale bars are 5 µm. **1f′–h′**. Three images of isolate CHINC-5 ATCC® 50979 (misidentified as *Sexangularia*) showing locomotive form. The absence of a shell, among other significant characters, indicates the identification as *Sexangularia* is incorrect. Note the finger-like pseudopods, similar to dactylopodids. Scale bars are 10 µm. Images of ATCC® isolates were generated by Jeffrey Cole and kindly provided by Robert Molestina, director of ATCC® collections, except for images on isolate CHINC-5 ATCC® 50979 provided by O. Roger Anderson.

**Figure 2:**
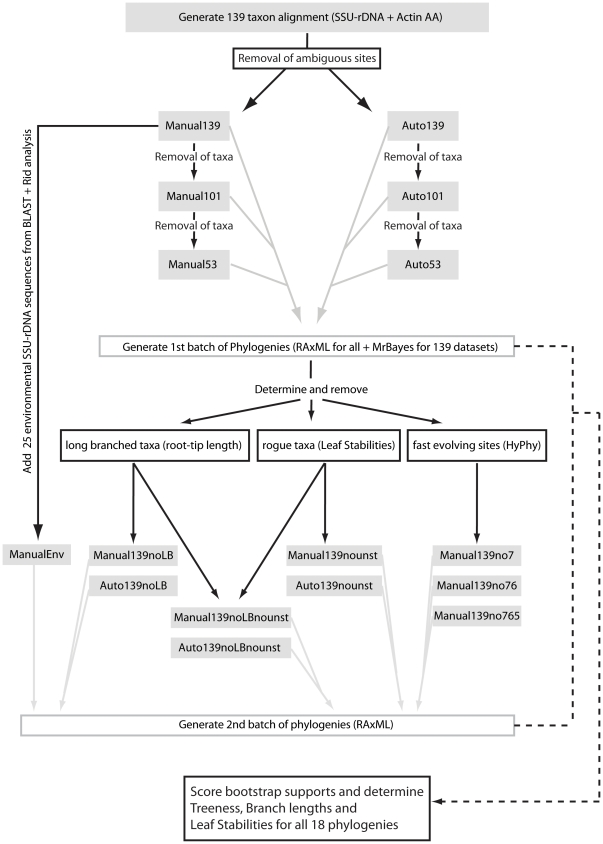
Computational pipeline implemented for phylogenetic analysis. Grey boxes indicate a dataset, grey arrows indicate phylogenetic analyses performed on that dataset. Black arrows and boxes indicate other types of analyses performed on particular datasets, and the black dotted lines indicate the final analyses performed to obtain scores for each phylogenetic reconstruction.

The SSU-rDNA and actin genes for 13 lineages were sequenced (Figure 1, Table 1) and phylogenetic analyses were performed on a total of 139 taxa (Supplementary Table S1), using multiple reconstruction strategies (Figure 2, Table 2). Topologies obtained in the 18 distinct phylogenetic reconstructions of concatenated SSU-rDNA and actin genes (Table 3, Figure S2) largely agree with previous analyses regarding the monophyletic status of lower-level relationships (defined here as in roughly equivalent to groups traditionally treated at the Order level or below in ranked classifications, Supplementary Figure S1). These groups are also consistent with morphological characters, as outlined in Smirnov et al. [15] and Shadwick et al. [28]: the Amoebidae, Dictyosteliida, dark spored myxogastrids, Hartmannellidae (excluding *Saccamoeba limax* ATCC® 30942), Leptomyxida, protosporangiids, protosteliids, schizoplasmodiids, soliformoviids and Tubulinida are always recovered with high support (Table 3); the Acanthamoebida, cavosteliids, Dactylopodiida, Echinamoeboidea, light spored myxogastrids, Mastigamoebida, Pelobiontida, Thecamoebida and Vannellida are recovered with moderate to high support; the Arcellinida are recovered with low support (Table 3).

**Table 1:** Newly characterized Amoebozoa lineages.

Taxon	Source	SSU-rDNA	Actin genes
*Cryptodifflugia operculata*	commercial culture	JF694280	JF694297-305
*Pyxidicula operculata*	Hiddensee Germany	JF694284	JF694316-318
*Arcella mitrata*	Hawley Bog, MA	JF694279	JF694293-296
*Arcella discoides*	Hawley Bog, MA	-	JF694287-292
*Arcella gibbosa*	Hawley Bog, MA	JF694278	-
*Hyalosphenia papilio*	Hawley Bog, MA	JF694282	JF694311
*Nebela carinata*	Hawley Bog, MA	JF694283	JF694312
*Gocevia fonbrunei*	ATCC® 50196	JF694281	JF694306-310
*Stereomyxa ramosa*	ATCC® 50982	-	JF694320-321
*Stygamoeba regulata*	ATCC® 50892	JF694285	JF694322
*‘Thecamoeba’* sp.	ATCC® 50185	JF694286	JF694323-326
*Paraflabellula hoguae*	ATCC® 30733	AF293899^a^	JF694313-315
CHINC-5 isolate^b^	ATCC® 50979	-	JF694319

a The SSU-rDNA for *Paraflabellula hoguae* ATCC® 30733 has been published previously [8]. We have obtained an identical sequence from our independently retrieved DNA.

b Morphological analysis confirms this isolate is not *Sexangularia*, mislabeled in the ATCC® collection.

Source indicates origin of the organism, GenBank numbers are listed for both SSU-rDNA and actin genes. Name in single quotes indicate that identification provided by ATCC® may be incorrect.

**Table 2:** Concatenated datasets generated to perform phylogenetic analyses.

Dataset name	Taxa #	Sites SSU-rDNA	Sites Actin	Removal of amb sites
A53	53	989	265	Automated
M53	53	1270	265	Manual
A101	101	989	265	Automated
M101	101	1270	265	Manual
A139	139	989	265	Automated
M139	139	1270	265	Manual
M139-7	139	1115	265	Manual
M139-76	139	1003	265	Manual
M139-765	139	860	265	Manual
A139-LB	129	1270	265	Automated
M139-LB	129	989	265	Manual
A139-us	129	1270	265	Automated
M139-us	129	989	265	Manual
A139-LB-us	120	1270	265	Automated
M139-LB-us	120	989	265	Manual
MEnv	164	1260	265	Manual

A list detailing which taxa were included in each reconstruction is available as Supplementary Table S1. Taxa # - number of taxa included in reconstruction; Sites – number of sites included in alignment for each of SSU-rDNA and actin genes; Removal of amb sites – method used for dealing with ambiguously aligned sites: Manual indicates that sites were hand curated, and Automated indicates usage of the GUIDANCE algorithm [79].

**Table 3:** Summary of bootstrap values obtained in all 18 reconstructions for previously proposed relationships and hypothesis suggested in the current report.

	A53	M53	A101	M101	A139	A139(B)	M139	M139(B)	M139-7	M139-76	M139-765	A139-LB	M139-LB	A139-us	M139-us	A139 -LB-us	M139-LB-us	MEnv
**Higher-level hypotheses**																		
Amoebozoa	92	89	84	90	81	1	85	1	90	49	nm	85	93	78	94	84	93	87
Myxogastrea (FD)	-	-	94	**96**	93	1	**96**	1	94	**97**	**95**	-	-	**97**	**97**	-	-	**95**
Tubulinea (S)	81	74	53	nm	66	0.79	60	0.84	54	59	26	66	65	72	55	67	75	4
Archamoebae (CS)	40	nm	nm	54	nm	nm	53	0.81*	55	55	nm	37*	61	nm	49	53*	45*	54
Mycetozoa (CS)	51	67	nm	nm	nm	nm	nm	nm	nm	nm	nm	-	-	nm	nm	-	-	nm
Flabellinea (S)	-	-	nm	nm	nm	nm	nm	nm	6	nm	nm	nm	nm	19	21	21	24	nm
Conosea (CS)	nm	nm	nm	nm	nm	nm	nm	nm	nm	nm	nm	nm	nm	nm	nm	nm	nm	nm
Discosea (CS)	-	-	nm	nm	nm	nm	nm	nm	nm	nm	nm	nm	nm	nm	nm	nm	nm	nm
Dermamoebida (CS)	nm	nm	nm	nm	nm	nm	nm	nm	nm	nm	nm	nm	nm	nm	nm	nm	nm	nm
Stelamoebea (CS)	-	-	nm	nm	nm	nm	nm	nm	nm	nm	nm	nm	nm	nm	nm	nm	nm	nm
Variosea (CS)	nm	nm	nm	nm	nm	nm	nm	nm	nm	nm	nm	nm	nm	nm	nm	nm	nm	nm
Varipodida (CS)	-	-	nm	nm	nm	nm	nm	nm	nm	nm	nm	nm	nm	nm	nm	nm	nm	nm
Protamoebae (CS)	nm	nm	nm	nm	nm	nm	nm	nm	nm	nm	nm	nm	nm	nm	nm	nm	nm	nm
**Proposed Hypotheses**																		
H1 ‘Poseidonida’	-	-	**100**	**100**	**100**	1	**100**	1	**100**	**100**	**100**	**100**	**100**	**100**	**100**	**100**	**100**	**100**
H2 ‘Fractovitellida’	-	-	**100**	**100**	**100**	1	**100**	1	**100**	**100**	**100**	**100**	**100**	**100**	**100**	**100**	**100**	**100**
H3 ‘Flamellidae’	-	-	57	82	nm	nm	76	0.89	nm	64	77	54	76	nm	69	52	75	67
H4 ‘Gracilipodida’	-	-	nm	nm	43	1	34	0.83	46	19	nm	48	41	40	41	42	50	24
H5 ‘Goceviidae’	-	-	-	-	90	1	83	0.88	88	76	34	**97**	**96**	-	-	-	-	80
H6 ‘Himatismenida+’	-	-	nm	nm	59	1	35	0.51	nm	44	18	49	30	-	-	-	-	28
**Morphogroups**																		
Amoebidae	**100**	**100**	**100**	**100**	**100**	1	**100**	1	**100**	**100**	**100**	**100**	**100**	**100**	**100**	**100**	**100**	**99**
Hartmannellidae**	**100**	**100**	**100**	**100**	**100**	1	**100**	1	**100**	**100**	**100**	**100**	**100**	**100**	**100**	**100**	**100**	79
Dictiosteliida	**100**	**100**	**96**	**96**	**97**	1	**97**	1	**96**	**96**	**98**	**97**	**97**	**100**	**100**	**100**	**100**	**96**
protosporangiids	-	-	-	-	**100**	1	**100**	1	**100**	**100**	**100**	**100**	**100**	**100**	**100**	**100**	**100**	**100**
DS Myxogastrea	-	-	**96**	**98**	**95**	1	**97**	1	**96**	**98**	**98**	**100**	**100**	**99**	**98**	**100**	**100**	**96**
soliformoviids	-	-	-	-	**100**	1	**100**	1	**100**	**99**	**97**	**100**	**100**	**100**	**100**	**100**	**100**	**100**
Leptomyxida	**100**	**100**	94	**96**	**98**	1	**96**	1	**96**	92	87	**97**	**95**	**99**	**97**	**99**	**96**	**96**
schizoplasmodiids	-	-	-	-	**100**	1	**100**	1	**99**	**97**	**96**	**100**	**100**	**99**	**100**	**100**	**100**	**97**
Cochliopodiidae	-	-	**100**	**100**	**100**	1	**100**	1	**100**	**100**	**100**	**100**	**100**	-	-	-	-	**100**
protosteliids	-	-	-	-	**100**	1	**100**	1	**99**	**98**	89	91	**96**	**100**	**100**	91	**98**	**100**
Tubulinida (Am+Hart)	**100**	**100**	80	69	75	1	82	1	86	92	nm	81	85	79	83	**97**	82	69
Dactylopodiida	-	-	-	-	**97**	1	92	0.**99**	80	47	36	45	90	**99**	**98**	**99**	**97**	92
Thecamoebidae	**96**	90	79	51	87	1	80	1	44	81	59	88	77	86	74	86	78	64
LS Myxogastrea	-	-	-	-	84	1	84	1	70	87	83	-	-	94	91	-	-	79
Echinamoeboidea	-	-	64	nm	73	0.**96**	77	0.**97**	83	70	44	75	81	75	80	80	84	70
Vannellida	**100**	**100**	**99**	**99**	68	1	54	0.**99**	42	36	nm	66	59	59	71	66	68	28
Centramoebida	-	-	58	42	77	1	73	1	33	30	nm	76	64	77	71	75	73	64
Mastigamoebidae	-	-	nm	39	nm	nm	66	0.58	71	77	59	nm	28	nm	64	nm	nm	59
Pelobiontidae	27	78	nm	36	nm	nm	58	0.91	71	78	13*	-	-	nm	56	-	-	56
cavosteliids	-	-	-	-	nm	nm	60	1	54	nm	nm	nm	37	nm	50	nm		51
Arcellinida	nm	nm	30	31	31	0.**95**	35	nm	27	nm	nm	33	32	37	36	36	34	2
*Sty*+*Ver*	-	-	nm	nm	nm	nm	nm	nm	nm	nm	nm	nm	nm	nm	nm	nm	nm	nm
Dic+pro	-	-	nm	54	nm	nm	69	1	62	47	45	nm	64	nm	78	nm	84	70

*denotes that the group is invaded by one incertae sedis taxon;

**excluding *Saccamoeba limax* ATCC® 30942.

All reconstructions performed on RaxML, except the two indicated by (B) on Mr. Bayes. Bootstrap and posterior probability values above 95 and 0.95 respectively are in bold. Notes: nm – non-monophyletic; - not enough taxa to test the group in reconstruction; DS Myxogastrea – dark spored myxogastrids; LS Myxogastrea – light spored myxogastrids; Am+Hart – Amoebidae+Hartmannellidae; *Sty*+*Ver* – *Stygamoeba+Vermistella*; Dic+pro – Dictyosteliida+protosporangiids; FD – taxon as defined in Fiore-Donno et al. [22]; S – taxon as defined in Smirnov et al. [15]; CS – taxon as defined in Cavalier-Smith et al. [14].

In contrast, almost all groups treated at ranks higher than Order in traditional classifications (Supplementary Figure S1) are not recovered in our analyses, with three exceptions (Table 3): 1) the Myxogastrea ( = myxomycetes) are recovered with high support in virtually all analyses, and both proposed nested groups are also strongly supported (dark spored myxogastrids and light spored myxogastrids); 2) the Tubulinea is recovered with moderate to high bootstrap supports in 15 out of 18 analyses, and 3 of the 4 group members Echinamoeboidea, Leptomyxoidea and Tubulinida are consistently recovered with moderate to high bootstrap supports. The fourth group, Arcellinida is recovered with low support in 13 out of 18 analyses. A further group within the Tubulinea (Hypothesis 1 – ‘Poseidonida’, see below) is highly supported in all analyses (Table 3); 3) the Archamoebae are recovered in 8 out of 18 analyses with weak to moderate support, the two proposed groups within are also moderately supported, the Pelobiontida is recovered with moderate to high support in 7 out of 14 analyses, and the Mastigamoebida in 8 out of 16.

Another two higher-level relationships worth noting are inconsistently recovered. The Mycetozoa *sensu* Cavalier-Smith *et al.* [14] (Myxogastrea+Dictyostellida+Protostellidae) are only recovered in analysis with low number of taxa included (Analyzes A53, M53 in Table 3). The Flabellinea are only recovered in analysis where long branched taxa and/or unstable taxa were removed (Table 3). All other proposed higher-level relationships are never recovered in our reconstructions: Flabellinea, Conosea, Discosea, Stelamoebea, Variosea and Varipodida (Table 3), but these are also not rejected using an AU test (Table 4).

**Table 4:** Summary of values obtained from approximately unbiased test.

Hypothesis tested	wkh	au	wsh
Conosa (CS)	0.153	0.185	0.632
Dermamoebida (CS)	0.354	0.482	0.893
Discosea (CS)	0.127	0.144	0.480
Flabellinea (S)	0.250	0.503	0.882
Glycosteliida (CS)	0.132	0.184	0.514
Macromycetozoa (FD)	0.254	0.450	0.806
Mycetozoa (CS)	0.130	0.250	0.669
Protamoeba (CS)	0.153	0.146	0.632
Stelamoebea (CS)	0.284	0.494	0.825
Variosea *sensu* (CS)	0.068	0.062	0.318
Varipodida *sensu* (CS)	0.254	0.423	0.794
*Stygamoeba*+*Vermistella*	0.253	0.387	0.743

Values are comparing our best phylogeny against phylogenies where proposed relationships were constrained. None of the hypotheses can be rejected, since all p values are above the 0.05 threshold. wkh – weighted Kishino-Hasegawa test; au – approximately unbiased test; wsh – weighted Shimodaira-Hasegawa test; FD – taxon as defined in Fiore-Donno et al. [22]; S – taxon as defined in Smirnov et al. [15]; CS – taxon as defined in Cavalier-Smith et al. [14].

### 2. Placement of newly characterized lineages

#### 2.1 Arcellinida lineages

**Figure 3:**
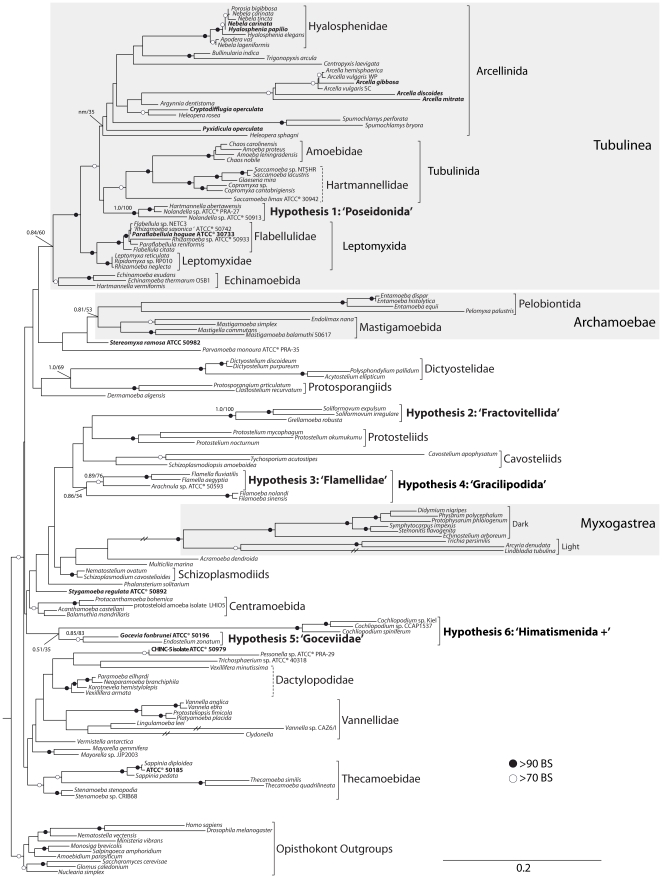
Phylogenetic reconstruction of the Amoebozoa, based on concatenated analysis of SSU-rDNA and actin genes of 139 lineages. This reconstruction is the best maximum likelihood tree obtained from the dataset Manual139, which we consider exhibits the optimal combination of tree indices and taxonomic coverage. Both Bayesian posterior probabilities and bootstrap supports are plotted on branches of interest. Branches without any support indication had bootstrap support of less than 70. The three well-supported higher-level groupings are shaded gray. The lower-level, morphologically consistent relationships are indicated. The novel relationships uncovered in the current study are in bold, and the suggested name for the group is shown in single quotes. Terminals in bold indicate lineages for which we are providing novel molecular information. Dashed brackets represent lower-level groups that are morphologically consistent but not recovered in this reconstruction. All branches are drawn to scale, except the branches leading to Myxomycetes, *Lindbladia*, *Vannella* CAZ6/I and *Clydonnella* which were trimmed to half-length for display purposes.

The newly introduced Arcellinida sequences consistently group with previously available lobose testate amoebae. The *Nebela carinata* and *Hyalosphenia papilio* fall consistently with other members of the Hyalospheniidae previously sequenced (Figure 3, Supplementary Figure S2). The three new lineages of the genus *Arcella* also consistently group with the other available *Arcella*, including *Arcella discoides* which is only represented by actin genes (Figure 3, Supplementary Figure S2). This demonstrates that at least in principle we should be able to infer relationships for the other two lineages represented only by actin genes (see below *Steromyxa ramosa* ATCC® 50982 and isolate CHINC-5 ATCC® 50979), as long as taxonomic sampling is significant. *Pyxidicula operculata* and *Cryptodifflugia operculata*, both representing previously unsampled genera, fall consistently in the Arcellinida, but with no consistent home (Supplementary Figure S2). The Arcellina hypothesis, which unites the testate amoebae that have secreted chitinous shells [35], would encompass the *Arcella*, *Pyxidicula* and *Spumochlamys*, but was not recovered.

#### 2.2 Other Amoebozoa lineages

The ATCC® accession 50196 identified as *Gocevia fonbrunei* is found to be strongly related to the protosteloid amoeba *Endostelium zonatum*. This relationship is moderately or highly supported in 9 out of 10 analyses where both taxa were present (Figure 3, Table 3). Further, *Gocevia fonbrunei*+*Endostelium zonatum* is monophyletic with *Cochliopodium* spp., albeit with moderate or low bootstrap supports in 9 out of 11 analyses where all taxa were present (Figure 3, Table 3). The ATCC® accession 50185, deposited as a member of the genus *Thecamoeba*, is nested within the genus *Sappinia*, with high support in all analyses (Supplementary Figure S2). *Sappinia* is in its turn the sister-group to the genus *Thecamoeba* [36], [37]. Analysis of the SSU-rDNA sequence performed by BLAST reveals that ATCC® 50185 is almost identical (99% similarity) to a specimen identified as *Sappinia* sp. Noaf (EU881941) [38], presumably related to *Sappinia diploidea*. This is an indication that isolate ATCC® 50185 is in fact a novel *Sappinia* lineage, and further research into its morphology should shed light on the distinctions between the two genera. The ATCC® accession 50892 identified as *Stygamoeba regulata*, and with morphological characters consistent with the original description [39] does not reliably fall into any of the proposed groups (Supplementary Figure S2). Leaf stability analyses do not indicate this as a particularly rogue taxon (Supplementary Table S2).

#### 2.3 Lineages represented only by actin genes

The two non-Arcellinida lineages for which we were only able to amplify the actin gene do not group reliably with any other Amoebozoa, which may either indicate their status as *incertae sedis* is granted, or that a single gene is not sufficient to reconstruct their evolutionary history. The ATCC® accession 50982 deposited as *Stereomyxa ramosa* does not reliably fall into any of the proposed groups, or lower-level morphological relationships (Supplementary Figure S2). In most reconstructions, it falls outside of the Archamoebae, but this is not supported by bootstrap analyses. The leaf stability index for this taxon is generally one of the lowest, ranking 26 out of 29 (29 being the most unstable taxa, Supplementary Table S2), further confirming its status as *incertae sedis* at least for this single gene. The isolate CHINC-5 ATCC® 50979 (misidentified as *Sexangularia* sp., see Material and Methods section) is found to be related to the also *incertae sedis Pessonella* sp., albeit with low bootstrap support (Supplementary Figure S2). Leaf stability analysis shows that both taxa are unstable, ranking 27 and 26 out of 29 (Supplementary Table S2).

### 3. Comparative analyses of different types of reconstruction

The general performance of 18 different reconstruction approaches was assessed by three measures: bootstrap supports of well-established morphological groups and proposed higher-level relationships (Table 3); leaf stability measures (Table 5, Supplementary Table S2); and Treeness indices (Table 5). Overall, trees tend to score higher with more taxa added; when manual removal of ambiguous sites is performed and when long branched as well as “rogue” taxa are removed (see Supplementary Text S1 for a detailed discussion). Since removal of 19 long branched or unstable taxa significantly impairs interpretation of relationships (for instance, Pelobiontida and Myxomycetes are almost completely removed), we consider that both Mr. Bayes and RaxML reconstructions based on the dataset with 139 taxa and manual removal of ambiguous sites (M139, Table 2) best represents our results (Figure 3), and comparisons will be made to other reconstructions as necessary.

**Table 5:** Summary of tree indices obtained for 16 RAxML reconstructions reconstructions.

Analysis	Tree Length	Treeness	LStability	95% CI
A53	9.47	0.35	0.84	0.02
M53	12.00	0.30	0.84	0.01
A101	21.66	0.36	0.82	0.02
M101	26.13	0.35	0.86	0.01
A139	31.48	0.40	0.80	0.01
M139	38.83	0.35	0.84	0.01
M139-7	21.05	0.34	0.86	0.01
M139-76	11.30	0.32	0.77	0.01
M139-765	7.78	0.32	0.73	0.01
A139-LB	26.77	0.45	0.80	0.01
M139-LB	30.73	0.41	0.85	0.01
A139-us	27.56	0.41	0.80	0.01
M139-us	34.36	0.37	0.85	0.01
A139-LB-us	24.14	0.45	0.83	0.01
M139-LB-us	27.76	0.41	0.88	0.01
MEnv	49.66	0.38	0.85	0.01

Tree length is the total length of the tree. Treeness index is the ratio of tree length that is in internal branches over the total tree length. Leaf Stability values are averaged over all taxa in 1000 boostrap reconstructions. The 95% Confidence Interval refers to Leaf Stability values.

### 4. Addition of environmental sequences

The addition of 25 environmental sequences neither improves support for the groups recovered in other reconstructions, nor stabilizes rogue taxa. The added sequences group with: Arcellinida (11 sequences), Mastigamoebidae (3 sequences), Hartmannellidae (2 sequences), undetermined (2 sequences) and one sequence in each *Cochliopodium*, Echinamoebidae, Filamoebidae, Myxogastrea, Poseidonidae, protosteliids and *Saccamoeba*. The bootstrap supports for lower-level relationships remain largely unchanged when compared to other types of reconstruction (Table 3); the average Leaf Stability is not significantly different from reconstructions with large taxon sampling (Table 5, Supplementary Table S2) and the Treeness index decreases when environmental taxa are added, probably the result of an increase in total tree length without a concomitant increase in signal (Table 5).

### 5. Actin gene family reconstruction

**Figure 4:**
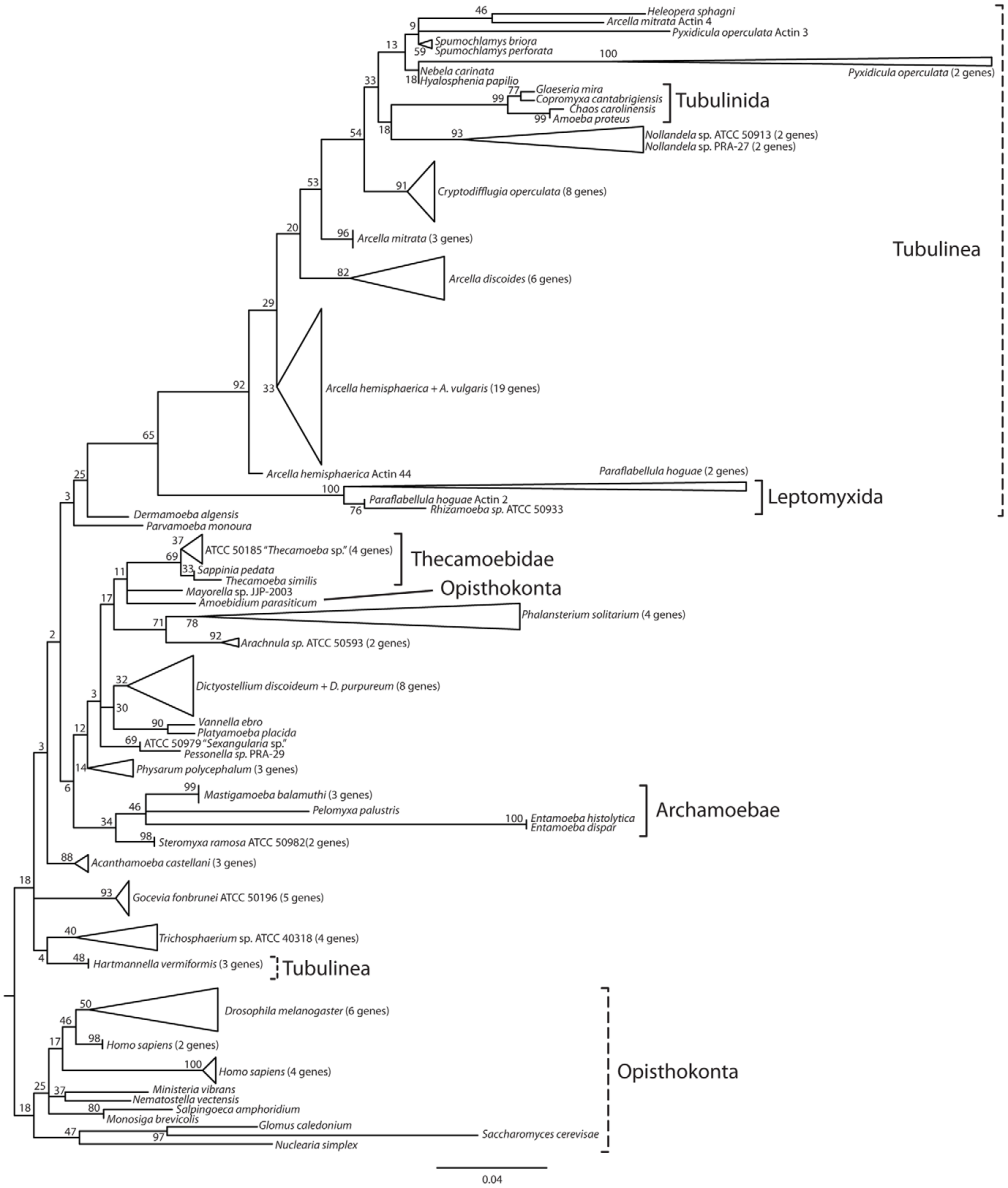
Reconstruction of actin gene family evolution in Amoebozoa, using 140 paralogs. Triangles indicate multiple paralogs (number indicated in parenthesis), the length of triangle is equal to the length of longest branching paralog within the group.

A reconstruction using multiple actin gene paralogs for 46 Amoebozoa taxa largely agrees with the reconstruction in Lahr et al. [40] (Figure 4). Using a reconstruction based on amino acids fails to recover monophyly of Amoebozoa, because under these conditions the Opisthokont *Amoebidium* does not fall as an outgroup. Still, many lower-level relationships are recovered (Figure 4): Leptomyxida, Tubulinida, Thecamoebidae, and one of the well-supported higher-level relationships is recovered: Archamoebae. However, the isolate *Hartmannella vermiformis* does not fall into the Tubulinea, another well-supported high-level relationship in the concatenated reconstruction. The Arcellinida appear as paraphyletic with the invasion of Tubulinida (Amoebidae+Hartmannellidae), indicating that some of the actin paralogs in these lineages may be ancient duplicates. Additionally, throughout the tree many taxa display recent independent expansions of the actin gene family (*Arcella*, *Cryptodifflugia*, *Dictyostelium*, *Phalansterium*, *Trichosphaerium*, *Gocevia*).

## Discussion

Our analyses of available SSU-rDNA and actin genes confirm the monophyly of several previously reported lower-level relationships (defined here as roughly equivalent to the Order level and below in traditional ranked systems) within the Amoebozoa, and indicate an additional six uncharacterized well-supported relationships (Figure 3, Table 3). However, only three of the previously proposed higher-level relationships (defined here as deep relationships that are above the level traditionally considered to be an Order) are consistently recovered: the Myxogastrea are strongly supported; the Tubulinea are moderately supported; and the Archamoebae are weakly supported. Other proposed higher-level relationships such as Conosea along with the included Mycetozoa and Archamoebae, as well as the Protamoebae with the included Discosea and Variosea are never recovered, but our data also do not reject these relationships (Table 4). The three recovered higher-level relationships are distinguished from other proposed groups in that they all have well-established morphological synapomorphies: the Tubulinea present cylindrical pseudopods with monoaxial streaming [15]; the Archamoebae unite all amitochondriate Amoebozoa (likely a secondary loss [41], rather than a primitive condition as previously suggested elsewhere [42]); and the Myxogastrea are characterized by a fruiting body arising from a coenocytic diploid stage [22], as well as ultrastructural details of biflagellate amoeboflagellates and plasmodial mitosis [14]. The remaining non-confirmed higher-level relationships (Table 3), which were proposed largely based on single gene analyses of SSU-rDNA, are not marked by strong morphological synapomorphies.

Most of the morphologically defined lower-level relationships are reliably recovered, as well as six previously undescribed groups, referred to here as Hypothesis 1–6 (Figure 3, Table 3). Proposed names for each hypotheses are stated in single quotes, to denote their speculative nature, and a taxonomy is provided for each group following regulations of the International Code of Zoological Nomenclature. The Tubulinea and nested groups are consistently well supported: Echinamoebida, Leptomyxida and Tubulinida are moderately to strongly supported and the Arcellinida is consistently recovered, albeit with weak support. Additionally Hypothesis 1 (‘Poseidonida’), a monophyletic group composed of *Nollandela* spp. and ‘*Hartmannella’ abertawensis* is distinct from the other four groups in the Tubulinea (Figure 3, Table 3). Indications of this relationship have been shown in previous reconstructions [22], [30], and our analysis suggests that this strongly supported group (Table 3) is not embedded within any other Tubulinea clade. *Nolandella* spp. and *‘Hartmannella’ abertawensis* were isolated from near-shore marine environments in the same publication [43]. Another species with similar morphological features, *Hartmannella vacuolata*, also marine, has been described with notes about the unusual feature for limax amoeba of a floating form with extended arms [44], a character shared with *Nolandella*. Both *Nolandella hibernica* and *Nolandella* sp. ATCC 50913 show the distinctive feature of a Golgi body nested within a concave portion of the nucleus (visible in figure 27 of [43] and figure 2A of [24]). Given the stable status of this clade, a unique shared morphological character, and the fact that the organisms share the marine environment as a habitat, we suggest the name ‘Poseidonida’, in reference to the Greek god of the seas, Poseidon (see taxonomic summary for a formal account). The type genus and species for the group should be *Nolandella hibernica* (Page 1980) for stability reasons, since *Hartmannella abertawensis* will likely require re-assignment to a new genus with further research [30].

The genus *Soliformovum*, common protosteloid amoebae found associated with dead plant material [45], forms a monophyletic group with *Grellamoeba robusta*, an amoeba isolated from fish kidneys [46], which we designate as Hypothesis 2 (‘Fractovitellida’, Figure 3, Table 3). *Grellamoeba robusta* is putatively related to *Acramoeba dendroida* based on SSU-rDNA analysis [46], which justified inclusion in the group Acramoebidae [47]. However Dykova et al. [46] emphasize that no well-supported relationships could be found in their analysis, either morphologically or phylogenetically, so they settled for an *incertae sedis* status. *Acramoeba dendroida* never groups with *G. robusta* in our analyses, suggesting *A. dendroida* is still the only representative of the Acramoebidae. On the other hand, *G. robusta* composes a new, highly supported clade with two soliformoviids (Hypothesis 2, see taxonomic summary for details). *Soliformovum* spp. was removed from the genus *Protostelium* based on a series of gross morphology and ultrastructural characteristics [48]. Spiegel et al. [48] suggests that the nucleus with an irregular, multilobed nucleolus is a putative synapomorphy of the genus *Soliformovum* (see Figure 16 and 18 in reference [48]), although the cavosteliid *Schizoplasmodiopsis amoeboidea* also presents a diffuse nucleolus [28], [49]. *Grellamoeba robusta* presents oval nucleoli more similar to *Protostelium* spp. and *S. amoeboidea* as a trophozoite, but shows a lobed morphology in cyst form (see Figures 11 and 13 in [46]), which may be consistent with the *Soliformovum* type [49]. The micrographs provided by Dykova et al. [46] do not indicate that *G. robusta* has a microtubular organizing center (MTOC), so this is possibly a further shared characteristic with the genus *Soliformovum* [48]. Both amoebae are generally uninucleate, without pigmentation and exhibit multiple contractile vacuoles. Gross-morphology is very similar, both present sharply pointed sub-pseudopodia and thus an acanthopodid morphotype (*sensu* Smirnov et al. [15]). However, *G. robusta* tends to be more branched and exhibit fan-shaped regions (see Figure 1 in [46]), while *Soliformovum*'s entire cell tends to be fan-shaped and less branched (see Figures 1–4 in [48]). No sorocarp formation was observed in *G. robusta* [46], making this novel relationship a suitable clade to further research the evolution of fruiting body formation in amoebae. We suggest this grouping be named ‘Fractovitellida’ (*fractus*-broken, *vitellum*-yolk) in reference to the diffuse aspect of the nucleoli (see taxonomic summary for a formal account), with type genus and species *Soliformovum irregularis* (Olive and Stoinanovich 1969).

Our analyses confirm the highly supported grouping of filopodia producing Amoebozoa in the genera *Flamella* and *Arachnula* sp. ATCC® 50593, which we designate as Hypothesis 3 (‘Flamellidae’, Figure 3, Table 3). *Flamella* are characterized by a fan-shaped morphology, with a wide anterior hyaloplasm that produces thin sub-pseudopodia and long trailing thin filipodia (see Figure 3 in [50]). Trophozoites present a central non-diffuse nucleolus, although *F. balnearia* shows an irregularly shaped nucleolus in the cyst form (see Figure 42 in [50]). Morphological information for *Arachnula* ATCC® 50593 reveals that it is a multinucleate amoeba with branched thin filopodia (see Figure 1D in [24]). This monophyletic relationship is within the moderately supported clade Hypothesis 4 (‘Gracilipodida’) as sister to *Filamoeba* spp., characterized by a flattened locomotive form with a thin anterior hyaloplasm and long, thin, filiform subpseudopodia [51], [52]. Hypothesis 4 has also been previously recovered, along with other environmental sequences [25], [50]. However, the previously proposed relationship between *Flammella* and *Acramoeba dendroida* is not recovered here [47]. Gross morphological features characterize Hypothesis 4 as outlined in Kudryavtsev et al. [50], but no putative ultrastructural synapormophies can be suggested at this point. The corroboration of both hypothetical clades in our analyses justify the designation of two nested amoeboid groups: Hypothesis 3 ‘Flammellidae’, containing *Flamella*+ATCC® 50593; and Hypothesis 4 ‘Gracilipodida’ (*gracilis*-slender, *pedes*-foot), in reference to the filose pseudopodia present in all members of the clade. The type genus and species for both groups is *Flammella magnifica* Schaffer 1926 according to the Principle of Priority (see taxonomic summary for a formal account).

The identification of ATCC® 50593 as *Arachnula* sp. in Tekle et al. [24] has been the subject of some controversy [53]. Bass et al. [53] suggest that large terminal fans provided with many thin reticulating pseudopodia, a conspicuous character in Cienkowski's description of *Arachnula* [54], are not present in the available images of isolate ATCC® 50593 [24]. Bass et al. [53] isolated an additional organism that they argue is more consistent with the original description [54]. In molecular analysis of SSU-rDNA, this organism falls in the Rhizaria along with other similar forms such as *Platyreta*. Bass et al. [53] then suggest that ATCC® 50593 is misidentified, and is instead more closely related to *Acramoeba dendroida* [47], but these do not group together in the current report. The isolate ATCC® 50593 instead is included in the well-supported clade of filopodia bearing Amoebozoa (Hypothesis 4 ‘Gracilipodida’) enforcing the notion that extremely similar, convergent morphologies are present in Amoebozoa and Rhizaria [53], corroboration based on molecular data is necessary to determine relationships. The taxonomic identity of *Arachnula* is further obscured because the organism in Figure 8 of Bass et al. [53] was unfortunately not amenable to culture (therefore cannot be studied further), and the authors themselves raise the possibility of contamination. Establishing a taxonomic identity by comparing traditional descriptions with modern techniques is a complicated affair [55], [56], and is made worse in this case by the multiple uncertainties introduced by different studies. The question of which organism is the real *Arachnula*, either ATCC® 50593 or the organism pictured in Figure 8 of Bass et al. [53] remains an open debate.

Hypothesis 5 (‘Goceviidae’) unites the amoeba *Gocevia fonbrunei* ATCC® 50196 and the protosteloid amoeba *Endostelium zonatum*, a relationship that has been previously suggested based on ultrastructure [57] and the common presence of a cell covering [45]. Although we present limited morphological data on ATCC® 50196, its morphology is generally consistent with that of *Gocevia fonbrunei* as having a lens-like locomotive morphology, few thin subpseudopodia and covered in a hyaline cuticle without foreign bodies and an unornamented cyst [2], [3], [58], [59]. The protosteloid amoeba *Endostelium zonatum* is characterized by a fibrous covering, and the amoeba has numerous thin subpseudopodia [60] (also see Figure 2i in [28]). The taxonomic status of this organism has been a conundrum, and has evaded classification in relation to other protosteloid amoebae [28], [41], [45], the very monophyly of the genus *Endostelium* has been called into question [45], despite evidence for the contrary [60]. The high stability of Hypothesis 5 enables us to suggest a novel Amoebozoa group, defined morphologically by the presence of an outer cuticle of fibrose or hyaline material. We suggest this group be named ‘Goceviidae’, the type genus and species should be *Gocevia fonbrunei* Pussard 1965 following the Principle of Priority. ‘Goceviidae’ is strongly supported and often recovered within a larger clade designated Hypothesis 6, along with the genus *Cochliopodium*, consistent with the ‘Himatismenida’ *sensu* Page [3], with the added inclusion of *Endostelium zonatum* (see taxonomic summary for a formal account). However, support is low for Hypothesis 6 and there is a chance that *Cochliopodium* spp. are grouping here due to a long-branch attraction artifact.

Our observation of a clade uniting the Dictyosteliida and the protosporangiids is inconsistent with previously published works. We do not recover the previously proposed Stelamoebea (Protostelida+Dictyosteliida) within the Mycetozoa (Stelamoebea+Myxogastrea) *sensu* [14]; nor the Macromycetozoa (Dictyostelidae+Myxogastrea) *sensu* [22], also observed in [25]; neither the grouping with cavosteliids [28]. However our analyses do not allow rejection of any of these hypotheses (Table 4). Given the moderate support for this clade, availability of equally likely alternative topologies, and lack of morphological features supporting any of these hypothesis, we suggest that the Dictyosteliida and the protosporangiids each be treated as groups of Amoebozoa *incertae sedis*.

Additionally, numerous taxa remain unplaced in our analyses: *Parvamoeba monoura*, *Stereomyxa ramosa*, *Dermamoeba algensis*, *Acramoeba dendroida*, *Multicilia marina*, *Phalansterium solitarium*, *Stygamoeba regulata*, ATCC® 50979, *Pessonella* sp., *Trichosphaerium* sp., *Vermistella antarctica* and *Mayorella* spp. are taxa with highly unstable relationships (Figure 3, Supplementary Figure S2). Morphological features of both *Vermistella antarctica* and *Stygamoeba regulata* would suggest these are closely related [39], [61], [62], but this relationship was not recovered (Table 3). However, AU testing does not reject a possible relationship (Table 4).

We hoped that including environmental sequences would increase resolution of the tree, a strategy previously adopted by several authors [14], [22], [25], [38], [47], [50]. However, the environmental sequences at most only add to already established morphological groups, and fail to resolve deep branches. This is corroborated by the low increase in the Treeness index, coupled with non-significant improvement in the average Leaf Stabilities (Table 5). We conjecture that obtaining phylogenetically meaningful SSU-rDNA sequences for amoebozoans from environmental surveys is an unreasonable expectation, given current technologies for environmental sampling of molecular sequences. SSU-rDNAs in Amoebozoa are often very divergent, exhibiting over 2,000 base pairs, and reaching 3,000–4,000 bp in some taxa (e.g. *Pelomyxa*, *Trichosphaerium*, *Lindbladia*). Additionally, many of these exhibit unusual secondary structure features [25]. In our experience, many amoeboid taxa are not easily amenable to routinely used molecular techniques, even the model organism *Dictyostelium discoideum* requires special techniques for reliable DNA preparation [63]. Key amoebozoan taxa likely have divergent SSU-rDNAs and will not be detected by current environmental sequencing methodology, but rather will need to be isolated and specifically targeted until better tools are developed for environmental sequencing. We provide in this paper two new methodologies that might simplify this task, by using single cell genome amplification as well as single cell cDNA extraction, while maintaining an acceptable morphological record through photodocumentation. These methods are superior to single cell PCR because they allow extraction of multiple genes from the same organism, crucial to the reconstruction of deep phylogenies.

What course of action should be taken to resolve the deep relationships within amoeboid organisms remains an open question. Our analyses demonstrate that single or few genes are not sufficient to uncover the relationships between deep groupings. Single gene analyses may however be enough to characterize relationships within well-supported lineages such as the Myxogastrea and Tubulinea. Morphological data are useful to establish synapomorphies among lower-level lineages, but also does not (at this point) help resolve the deeper relationships. Whether phylogenomic approaches (analyzing alignments of entire genomes) hold the key to resolve these ancient relationships remain to be seen. It is not clear as yet that such analyses actually result in more signal or yield strongly supported biased answers [64], another option may be using a selection of well chosen genes as in Parfrey *et al.* [65]. Unrestrained proposition of novel higher-level taxa in Amoebozoa based on single gene analyses is a practice that should be avoided. Given the considerable uncertainty within Amoebozoa phylogeny, we encourage future authors to be cautious when proposing taxon names based on poorly supported groups, even if these are not rejected by AU tests—a statistical test is only as powerful as the dataset allows the test to be. Thorough phylogenetic analyses with comprehensive taxon sampling, multiple genes and ideally compelling morphological characters are a necessity before any new taxa be proposed.

An additional important factor in disentangling the phylogenetic relationships within the Amoebozoa is comprehensive taxon sampling. The recognition that protosteloid amoebae are an integral part of the Amoebozoa [28], [30] opens up many possibilities for exploring possible taxa with key phylogenetic positions, as suggested by the stabilization of three homeless amoeboid taxa (*Gocevia fonbrunei*, *Cochliopodium* sp. and *Grellamoeba robusta*) due to inclusion of protosteloid amoebae in our analyses (Figure 3, Table 3). This integration will most likely be useful not only in phylogeny, but also allow more meaningful studies on several aspects of Amoebozoa evolution, such as the evolution of the many diverse life cycle strategies [66].

### Taxonomic summary of hypothesis proposed in the current report

#### Remarks on nomenclature

At the time of writing of this report, there is no widely agreed upon consensus on how microbial eukaryote taxa should be named and treated. Some advocate a rankless approach while others continue to propose categorical ranks along with their taxon names. The International Code of Zoological Nomenclature, International Code for Botanical Nomenclature and the Bacterial Code do not assume direct responsibility for new microbial eukaryote names, they merely suggest ways to deal with names that were originally described under their provisions. We have taken a pluralistic approach with the aim to stabilize and make the taxa we propose available under many circumstances. We suggest taxa under categorical ranks, but those who wish to create a rankless taxonomy are welcome to ignore the proposed ranks, and be guided by the Hypotheses in Figure 3. Names are suggested in accordance with the ICZN: we provide diagnosis, etymology and name-bearing types. Additionally, we provide putative synapomorphies (where possible), which are not required by the ICZN.

Phylum Amoebozoa Luhe, 1913

 Class Tubulinea Smirnov et al. 2005

  Order Poseidonida ord. nov. Lahr and Katz 2011

   Diagnosis: marine limax amoebae; small (5–20 um); pseudopods with a cylindrical or semi-cylindrical cross-section and monoaxial streaming; Golgi body nested within a concave portion of the nucleus.

   Type species: *Nolandella hibernica* (Page 1980)

   Etymology: in reference to the Greek god of the seas, Poseidon. All organisms in this group are marine, or capable of tolerating high-levels of salinity.

   Putative Synapomorphy: marine limax Tubulinea

    Family Nolandellidae fam. nov. Lahr and Katz 2011

     Included taxa: *Nolandella*; *‘Hartmannella’ abertawensis*.

     Diagnosis: with characters of the order Poseidonida.

     Type species: *Nolandella hibernica* (Page 1980)

     Etymology: in direct reference to the type species.

 *Incertae sedis* Amoebozoa

  Order Fractovitellida ord. nov. Lahr and Katz 2011

   Diagnosis: uninucleate amoebae without coloration, irregularly triangular with sharply pointed hyaline sub-pseudopodia, lobed nucleoli, and absence of a microtubular organizing center (MTOC).

   Type species: *Soliformovum irregularis* (Olive and Stoianovich 1969) Spiegel 1994

   Etymology: From the Latin fractus (broken) and vitellum (yolk), in reference to the appearance of the nucleoli. Also to acknowledge the etymology of the genus *Soliformovum*, which alludes to the resemblance of the pre-spore to a fried egg “sunny-side up” (Spiegel et al. 1994).

   Putative Synapomorphy: presence of lobed nucleoli in at least one stage of the life-cycle.

    Family Soliformoviidae fam. nov. Lahr and Katz 2011

     Included taxa: *Soliformovum*, *Grellamoeba*

     Diagnosis: with characters of the order Fractovitellidae.

     Type species: *Soliformovum irregularis* (Olive and Stoianovich 1969) Spiegel 1994

     Etymology: in direct reference to the type species.

  Order Gracilipodida ord. nov. Lahr and Katz 2011

   Included taxa: Flamellidae fam. nov. Lahr and Katz 2011, Filamoebidae Cavalier-Smith 2004

   Diagnosis: gross morphological features outlined in Kudryavtsev 2009: flattened locomotive form either with expanded fan-shaped hyaloplasm regions producing thin sub-pseudopodia, or pseudopods coming out from cell body. Pseudopods are thin, filiform. Single or multinucleated.

   Type species: *Flamella magnifica* Schaeffer 1926

   Etymology: from the Latin gracilis (slender) and pedes (feet), in reference to the ability shared by these organisms to produce thin pseudopodia.

   Putative Synapomorphy: filiform pseudopodia.

    Family Flamellidae fam. nov. Lahr and Katz 2011

     Included taxa: *Flammella*, *Arachnula* ATCC® 50593

     Diagnosis: flattened, sometimes fan-shaped amoebae that can produce digitiform sub-pseudopodia from an anterior wide hyaloplasm margin, or can produce thin pseudopods from the cell body. Central, non-diffuse nucleolus in trophozoites.

     Type species: *Flamella magnifica* Schaeffer 1926

     Etymology: in direct reference to the type species, and most well described genus.

  Order Himatismenida Page 1987 emend.

   Diagnosis: amoebae with a locomotive lens-like shape, with an organic covering that does not enclose the cell completely, and may be organized in scales.

   Type species: *Cochliopodium bilimbosum* Auerbach 1856

   Putative Synapomorphy: an organic outer covering which does not completely seal the amoeba.

    Family Cochliopodidae Hertwig and Lesser 1874 emend.

     Included taxa: *Cochliopodium*

     Diagnosis: himatismenid amoebae capable of producing an organic tectum composed of structured scales.

     Type species: *Cochliopodium bilimbosum* Auerbach 1856

     Putative Synapomorphy: structured scales composing the outer covering.

    Family Goceviidae fam. nov. Lahr and Katz 2011

     Included taxa: *Gocevia*, *Endostelium*

     Diagnosis: himatismenid amoebae capable of producing non-organized outer cuticle, hyaline or granular.

     Type species: *Gocevia fonbrunei* Pussard 1965

     Etymology: in direct reference to the type species.

     Putative Synapomorphy: an outer cuticle made of non-structured organic material.

## Materials and Methods

### 1. New taxa and morphology

Molecular sequences of SSU-rDNA and/or Actin were generated for 13 taxa (Table 1, Fig. 1). The testate amoeba *Cryptodifflugia operculata* (Figure 1a–c) was isolated from a mixed Protozoa culture (Carolina Biological Supply Company, Cat. No. 131970). *Arcella mitrata* (Figure 1d–f), *Arcella gibbosa* (Figure 1g–i), *Arcella discoides* (Figure 1j–l), *Hyalosphenia papilio* (Figure 1s, t) and *Nebela carinata* (Figure 1z,a′) were isolated from *Sphagnum* sp. moss in Hawley Bog, MA. *Pyxidicula operculata* (Figure 1m, n) was isolated from Hiddensee, Germany and kindly donated to us by Mr. Wolfgang Bettighofer. *Gocevia fonbrunei* ATCC® 50196 (Figure 1o–r), *Stereomyxa ramosa* ATCC® 50982 (Figure 1u–y), *Stygamoeba regulata* ATCC® 50892 (Figure 1b′–e′), isolate CHINC-5 ATCC® 50979 (Figure 1f′–h′), *Thecamoeba* sp. ATCC® 50185, *Paraflabellula hoguae* ATCC® 30733 were obtained from the American Type Culture Collection (Manassas, VA).

All ATCC® species were identified following the original depositor information, and, when possible, comparison of photodocumentation provided by ATCC® to the original literature. We maintained the original depositor identification for all organisms except isolate CHINC-5 ATCC® 50979, which is certainly not a *Sexangularia* since it does not possess a shell (**Figure 1f′–h′**). This organism presents morphological characteristics similar to the dactylopodids, and will be described in detail elsewhere. The accuracy of the original identification for all other accessions will be discussed further after molecular analyses. However complete morphological characterization of these isolates is outside the scope of the current essay, and only limited morphological conclusions will be drawn.

The Arcellinida were identified by light microscopy and scanning electron microscopy where necessary (for electronic microscopy methods, see [67]). We established a clonal culture of *Cryptodifflugia operculata*, whose morphological characteristics are in accordance with the original description [68], including the presence of a mucous aperture plug after encystation (operculum, Figure 1a). The operculum is regarded as the only distinguishing characteristic between *C. operculata* and the type species *C. oviformis* Penard 1890, and its use as a distinguishing character has been challenged as it may vary intra-specifically [69]. We use Page's *C. operculata* definition since the operculum has indeed been observed in our isolate, and further research on non-operculum forming lineages is needed to elucidate this issue. Our clonal culture of *Pyxidicula operculata* had morphological characteristics in accordance with those described in Cash et al. [70]. Some individuals presented a small funnel shaped rim attached to the inner side of the shell that is characteristic of *Pyxidicula patens* (Claparede and Lachmann 1859) indicating that the character may vary intra-specifically. The three *Arcella* isolates were identified in accordance with appropriate literature [55], [71], *Arcella discoides* and *Arcella gibbosa* were culturable, while one *A. mitrata* individual was isolated from nature, photodocumented and genome amplified (see section 2). *Hyalosphenia papilio* and *Nebela carinata* individuals were isolated from nature, photodocumented and further processed (section 2), morphological characteristics in accordance with those of Lara et al. [18].

### 2. Molecular methods: DNA extractions, primers used, PCR conditions, cloning

A combination of multiple methods was used to characterize both SSU-rDNA and actin genes from the diverse lineages. The ATCC® samples were processed as described in Tekle et al. [24]. Briefly, cultures were harvested and DNA extracted using DNA Stat60 (Tel-Test, Inc., Friendswood, Texas, Cat. No. TL-4220) following manufacturer's instructions, with the addition of a Phenol-Chloroform-Isoamyl step using Phase Lock Gel Heavy tubes (Eppendorf AG, Hamburg, Germany, Cat. No. 955154070).

We used multiple strategies for obtaining DNA from the testate amoebae species, due to their resistance to PCR methods and the difficulty in culturing some species. *Arcella gibbosa*, *Arcella discoides*, *Pyxidicula operculata* and *Cryptodifflugia operculata* were cultured in autoclaved pond water enriched with cereal grass media extract and bacteria as described in [40]. DNA was extracted using a standard Phenol:Chloroform protocol on rapidly growing cultures as in [40], [72]. *Arcella mitrata*, *Nebela carinata* and *Hyalosphenia papilio* were not amenable to culture, so we adopted two alternative strategies before PCR: whole genome amplification and cDNA extraction of single individuals. Briefly, for both strategies, a single or a small group of individuals were cleaned through several sterile pond water washes, left overnight to purge any remaining food/prey organisms being digested, re-washed in sterile pond water, and photo-documented in a light microscope. The individuals were then placed in either buffer DLB from a Repli-g Mini Kit (Qiagen, Cat. No. 150023) for whole genome amplification, or in Resuspension buffer with Lysis Enhancer from a SuperScript III CellsDirect cDNA synthesis kit (Invitrogen, Cat. No. 18080-200). Genome amplification and generation of complementary DNA libraries were then performed following manufacturer's instructions. PCR reactions on obtained DNAs were tested on a serial dilution (1x-1∶1000 in ddH_2_O), and the lowest concentration amplification was chosen to avoid formation of chimeras for further processing [72]. Using this strategy enables a similar comparison to clonal cultures, since we have obtained the genetic material from a single individual. Primers for SSU-rDNA amplification were from [73] with three additional primers used to generate overlapping sequences from each clone [74], or shorter internal sequences for organisms where full SSU-rDNA amplification was not possible. Primers for actin were from [75] and [72]. Phusion Hot Start DNA polymerase (New England BioLabs, Cat. no. F540) was used to amplify the genes of interest, and Zero Blunt TOPO cloning kits (Invitrogen, Cat. No. K280020) were used to clone PCR products. Cloned plasmid DNA was purified in a 96 well format using a PureLink Kit (Invitrogen, Cat. No. 12263018) and sequencing reactions performed using an ABI3100 sequencer (Applied Biosystems, Foster City, CA, USA) either at the Smith College Center for Molecular Biology (Northampton, MA) or at the Pennsylvania State Nucleic Acid Facility (University Park, PA, USA).

### 3. Multiple Sequence Alignments

#### 3.1. SSUrDNA datasets

Sequences for SSU-rDNA of 117 Amoebozoa and 10 Opisthokonta outgroups were retrieved from GenBank (Supplementary Table S1), along with the 9 SSU-rDNA sequences generated in this study (Table 1) for a total of 136 SSU-rDNA sequences. Taxon sampling reflects an effort to include representatives of all available lineages in the ‘Amoebozoa’ [1], [13], [23], [24], [28]. Alignments were constructed in SeaView [76], [77] with alignment algorithm MAFFT [78] using the L-INS-I setting. Alignments were then curated manually to adjust ambiguous regions. This larger alignment was then subject to manual removal of ambiguous sites, to generate the dataset named M139 (Figure 2, Table 2). Independent automated removal of ambiguous sites was done using the online server GUIDANCE [79] with default parameters, to generate the dataset named A139 (Figure 2, Table 2).

Additional datasets with limited number of taxa were generated to explore the interaction between taxon sampling and missing actin sequences (Figure 2, Table 2). We removed taxa from both A139 and M139 to contain at least one representative of each major lineage, while maintaining all taxa for which actin sequences are available (43), to a total of 101 taxa, generating thus the alignments names A101 and M101 (Figure 2, Tables 2, see Supplementary Table S1 for a detailed list of taxon inclusion). Both datasets were subjected to further taxon removal to maintain only the 43 Amoebozoa lineages for which both actin and SSU-rDNA sequences are available as well as the 10 outgroup sequences, generating datasets A53 and M53 (Figure 2, Table 2, Supplementary Table S1). Datasets were then concatenated with the amino-acid actin dataset obtained in section 3.2 and subject to post-phylogenetic analyses treatment, as explained in Section 4.1.

#### 3.2. Actin datasets

Representative sequences for actin genes of Amoebozoa were retrieved from GenBank, curated Genome databases and EST databases, as detailed in [40]. The dataset, containing a total of 130 actin genes, 40 of them generated in this study (13 taxa, some with multiple paralogs, Table 1), was aligned at the amino-acid level in the software SeaView [77] using the alignment algorithm MAFFT [78] set to L-INS-I optimization, and trimmed down to retain only a central homologous region. The dataset for actin consists of 130 sequences with 265 amino acid sites. To choose sequences for concatenation, we determined the shortest branched actin genes for each group of paralogs, through a PhyML [80] analysis using a GTR model, with optimized estimation of invariable sites, gamma variation with 6 rate categories across sites, combining the best of NNI and SPR searches, as implemented in Seaview [77]. We then trimmed the alignment to contain only the shortest branched paralog for each species, totaling 46 Amoebozoa taxa, and 265 amino-acid sites. This dataset was then concatenated to six SSU-rDNA datasets obtained in section 3.1 (A139, M139, A101, M101, A53 and M53). Additionally the alignment with all 130 paralogs was analyzed separately to determined events in the evolution of actin gene families in Amoebozoa. We performed maximum likelihood analyses on the amino acid dataset as described in section 4.1.

### 4. Phylogenetic analyses

#### 4.1. Concatenated datasets

We performed maximum likelihood phylogenetic reconstruction in each of the initial six concatenated datasets using RAXML HPC 7.2.7 [81], [82] as implemented in the online server CIPRES [83]. We ran 1000 fast bootstrap analysis using the GTRCAT approximation, and 100 independent maximum likelihood reconstructions using the GTRGAMMA model for the SSU-rDNA partition and the LG model for the protein partition. The most appropriate model for amino-acid evolution was determined using model testing implemented in the online server Datamonkey [84]. Bootstrap values of the GTRCAT search were then plotted on the best tree found by maximum likelihood search for comparative analysis. Additional Mr. Bayes analyses were performed on the two largest datasets Auto139 and Manual139 to test independence of results from algorithm. We used the implementation on BioHPC cluster at Cornell University (http://cbsuapps.tc.cornell.edu/). Using a random starting tree, the analyses did not converge after 20 million generations. Because Mr. Bayes is so computationally heavy, we had to resort to starting analyses from the best ML tree obtained in RAXML, although this may lead to exaggeration of support values in the final Bayesian tree. Hence, we started the analysis using the topology obtained in the RAxML analysis, using the npert command to disturb the initial tree and avoid biasing results. The analysis was run for 10 million generations, saving trees every 2000 generations. We performed two independent MCMC runs with 8 chains each, and a heating parameter of 0.05. We obtained convergence after 4 million generations, the 2,000 trees before convergence were discarded as burnin and analyses were made on the remaining 3,000 trees. We applied the GTR+gamma model for the SSU-rDNA partition, and the WAG model for protein partition, since the available version of Mr.Bayes did not implement the LG model at the time of writing this report. The WAG model was the second best fit to our data according to the model selection analysis performed in the online server Datamonkey.

#### 4.2. Removal of fast rate sites, long-branched and unstable taxa

To assess the effect of rate heterogeneity on SSU-rDNA topologies, we partitioned the Manual139 dataset into 8 rate classes using the GTR model with rate variation among sites following a discrete gamma distribution, as implemented in HyPhy v1.0beta [85]. Classes 0 and 7 represent the slowest and fastest rate classes, respectively. We then proceeded to eliminate the fastest rate class (7) to generate the alignment M139-7 (Table 2). Similarly, we removed the two fastest rate classes (7 and 6) for the dataset M139-76, and the three fastest rate classes (7, 6 and 5) for the dataset M139-765 (Table 2).

To assess the effect of long-branched taxa on final topologies the root-tip branch lengths of each terminal from section 4.1 was calculated as implemented in the freely distributed program TreeStat v1.2 (http://tree.bio.ed.ac.uk/software/treestat/). The results were then compared within reconstructions and we proceeded to remove the 10 overall longest branched taxa (*Arcyria denudata*, *Didymium nigripes*, *Echinostelium arboreum*, *Lindbladia tubulina*, *Pelomyxa palustris*, *Physarum polycephalum*, *Polysphondylium pallidum*, *Protophysarum phloiogenum*, *Trichia persimilis* and *Tricosphaerium* sp. ATCC 40318, a list of all Branch lengths is available as Supplementary Table S3), to generate the alignments M139-LB and A139-LB, with a total of 129 taxa each (Figure 2, Table 2).

To assess the effect of unstable taxa on final topologies we calculated terminal Leaf Stabilities [86] as implemented by the script THOR (http://code.google.com/p/phylogenetics/) using as input the outgroup-rooted 1000 bootstrap trees generated from Section 4.1. After performing comparative analysis between the different datasets, we removed the 10 most unstable taxa (the three *Cochiopodium* spp., *Dermamoeba algensis*, *Endostelium zonatum*, *Gocevia fonbrunei* ATCC 50196, *Pessonella* sp., isolate CHINC-5 ATCC 50979, *Trichosphaerium* sp. and *Vexilifera minutissima*) to generate the datasets M139-us and A139-us, with a total of 129 taxa each (Figure 2, Table 2). Additionally, we generated datasets by removing both the most unstable taxa and the most long-branched taxa at the same time, to a total of 120 taxa in the dataset A139-LB-us and M139-LB-us (Figure 2, Table 2).

#### 4.3. Sampling of environmental sequences

A next logical step for our analyses was to determine whether increased taxon sampling will enable more robust phylogenetic reconstructions. An available method widely used to increase taxon sampling is to add environmental sequences that represent unculturable organisms or taxonomic representatives in environments that were not yet studied by specialists. The number of environmental sequences available is very large, and there is a tendency to recover closely related organisms since most environmental studies are focusing on a specific type of habitat, rather then targeting phylogenetic coverage. It is desirable then to use representatives from different parts of the tree rather than many representatives in a single branch (cherries). We performed BLAST searches querying all 129 Amoebozoa taxa in our dataset against the environmental database in GenBank. We retrieved the top 100 hits for each taxon to create a combined dataset, excluding redundant sequences of ∼3,000 entries. We then eliminated all entries that are 98% similar to each other using the Rid v0.3 script (Grant, J.). This approach recovered 25 sequences that were then included in the M139 datasets, generating the dataset MEnv (Figure 2, Table 2).

#### 4.4. Comparative analyses of resulting trees

We used three methods to assess the information in our reconstructions: comparison of bootstrap supports for different levels of groupings, Treeness Index and Leaf Stabilities. For comparative analysis of support for different groupings, we divided the hypothesized groupings in two categories: higher-level relationships and morphology based lower-level relationships. We then assessed bootstrap supports from the 18 reconstructions performed to compare stability of clades between analyses. We also compared data for the Treeness index, a measure of the proportion of total tree length that is taken up by internal branches, thought to be a rough assessment of how much of the dataset's information is being used to reconstruct stem relationships as opposed to substitutions along terminal branches. We calculated Treeness values as implemented in TreeStat (http://tree.bio.ed.ac.uk/software/treestat/). Finally, we calculated the average leaf stability for each reconstruction; this is useful in informing how much overall instability is present in a particular dataset, and whether our manipulations are improving overall resolution.

### 5. Approximately unbiased (AU) testing of alternative hypotheses

We tested whether non-recovered hypotheses could be rejected using the Approximately Unbiased test [87]. Briefly, we generated maximum likelihood reconstructions with constraints for each of 12 alternative hypotheses by running 100 independent maximum likelihood analysis in RAxML using the exact same parameters as before, and choosing the most likely tree. All trees were then compared to the best tree found on the standard analysis using RAxML to calculate per-site likelihoods. The per-site likelihoods were then analyzed in CONSEL [88] with standard parameters to obtain p-values.

## Supporting Information

Figure S1 A summary of previously proposed relationships between the Amoebozoa.
(TIF)

Figure S2 Diagrams of all 18 reconstructions performed.
(PDF)

Table S1 List of organisms, abbreviations and accession numbers for SSU-rDNA and actin genes used in concatenated analyses. Each organism is specified as to wheater it was included or not in a particular set of analyses with an x.
(XLS)

Table S2 Leaf Stability values obtained for each taxon in each different reconstruction. Taxa are ranked by their average Leaf Stability.
(XLS)

Table S3 Root to tip branch lengths for each taxon in each reconstruction. Taxa are ordered from shorter to longer average branch lengths.
(XLS)

Text S1 (DOC)
