# Supplementary figures and images for: Comprehensive Phylogenetic Reconstruction of Amoebozoa Based on Concatenated Analyses of SSU-rDNA and Actin Genes
Source: PLoS One. 2011 Jul 28;6(7):e22780. doi: 10.1371/journal.pone.0022780 (PMC3145751; doi:10.1371/journal.pone.0022780)

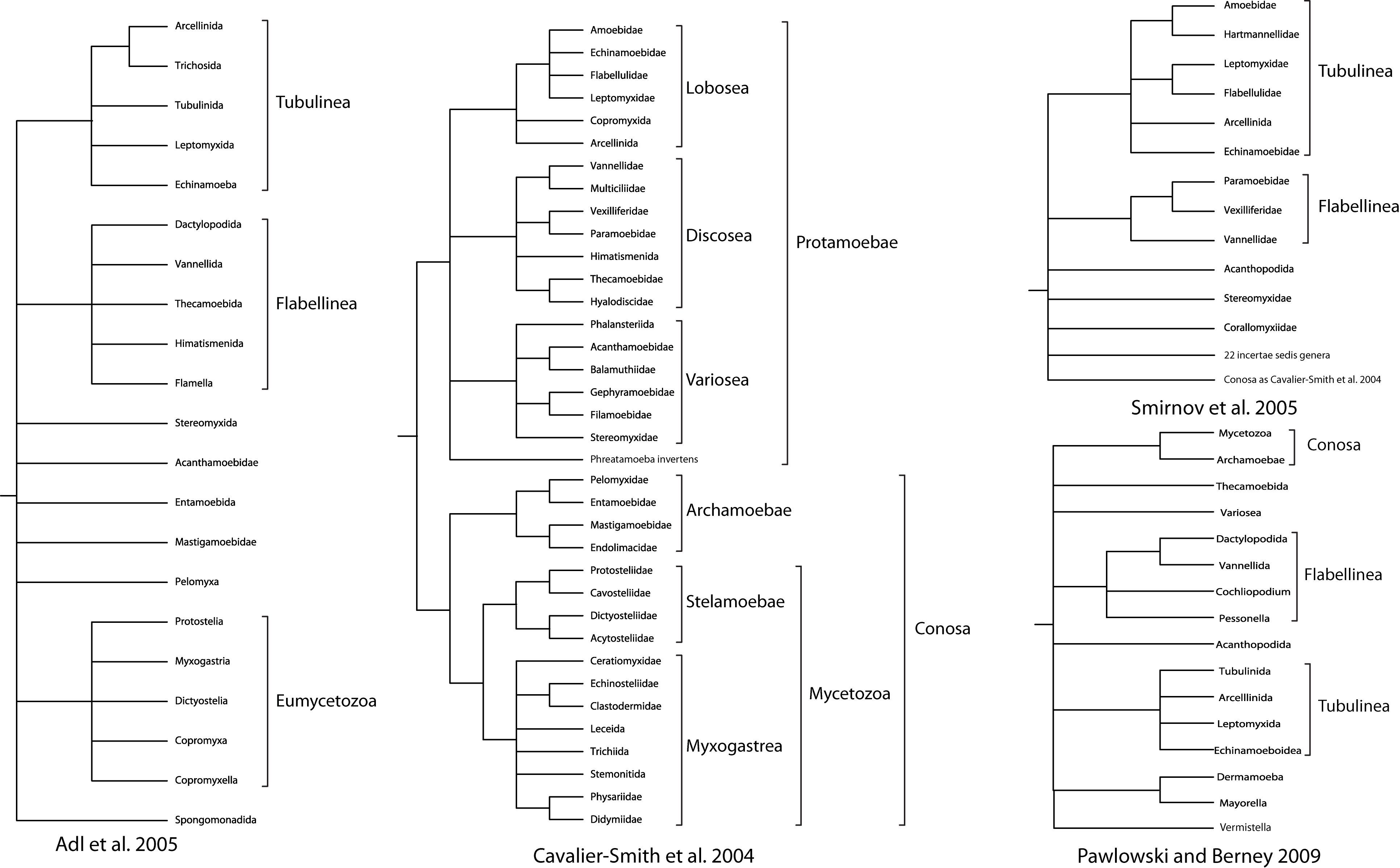

Supplement: Figure S1 — A summary of previously proposed relationships between the Amoebozoa. (TIF) [file pone.0022780.s001.tif]
