# Supplementary material for: Comprehensive Phylogenetic Reconstruction of Amoebozoa Based on Concatenated Analyses of SSU-rDNA and Actin Genes
Source: PLoS One. 2011 Jul 28;6(7):e22780. doi: 10.1371/journal.pone.0022780 (PMC3145751; doi:10.1371/journal.pone.0022780)
Supplement: Figure S2 — Diagrams of all 18 reconstructions performed. (PDF) [file pone.0022780.s002.pdf]

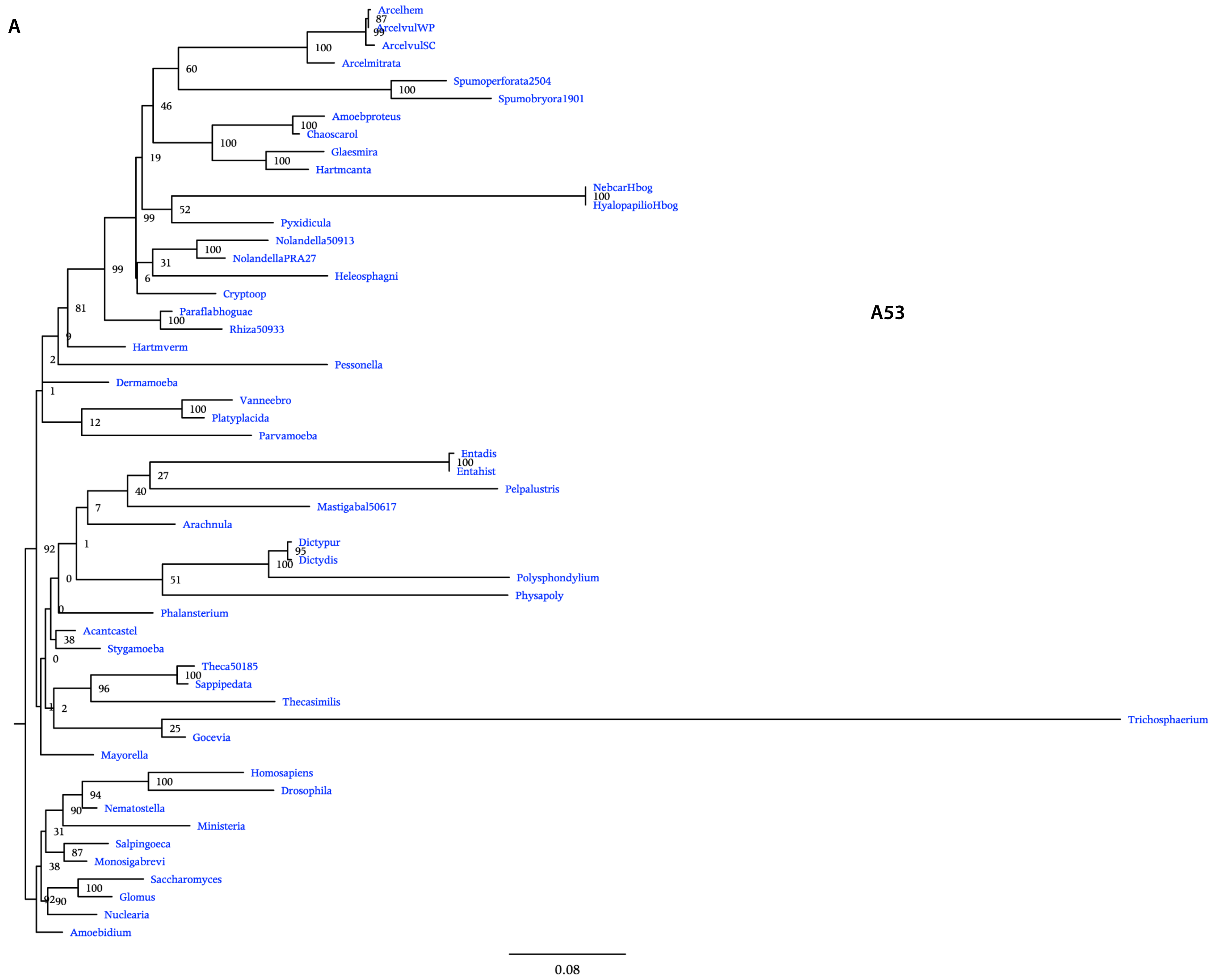

A
A53
Arcelhem
87
ArcelvulWP
99
ArcelvulSC
100
Arcelmitrata
60
Spumoperforata2504
100
Spumobryora1901
46
Amoebproteus
100
Chaoscarol
100
Glaesmira
100
Hartmcanta
19
NebcarHbog
100
HyalopapilioHbog
52
Pyxidicula
99
Nolandella50913
100
NolandellaPRA27
31
Heleosphagni
6
Cryptoop
99
Paraflabhoguae
100
Rhiza50933
81
9
Hartmverm
2
Pessonella
Dermamoeba
1
Vanneebro
100
Platyplacida
12
Parvamoeba
Entadis
100
Entahist
27
Pelpalustris
40
Mastigabal50617
7
Arachnula
92
1
Dictypur
95
Dictydis
100
0
51
Polysphondylium
Physapoly
0
Phalansterium
Acantcastel
38
Stygamoeba
0
Theca50185
100
Sappipedata
96
Thecasimilis
1
2
Trichosphaerium
25
Gocevia
Mayorella
Homosapiens
100
Drosophila
94
Nematostella
90
Ministeria
31
Salpingoeca
87
Monosigabrevi
38
Saccharomyces
100
Glomus
92
90
Nuclearia
Amoebidium
0.08

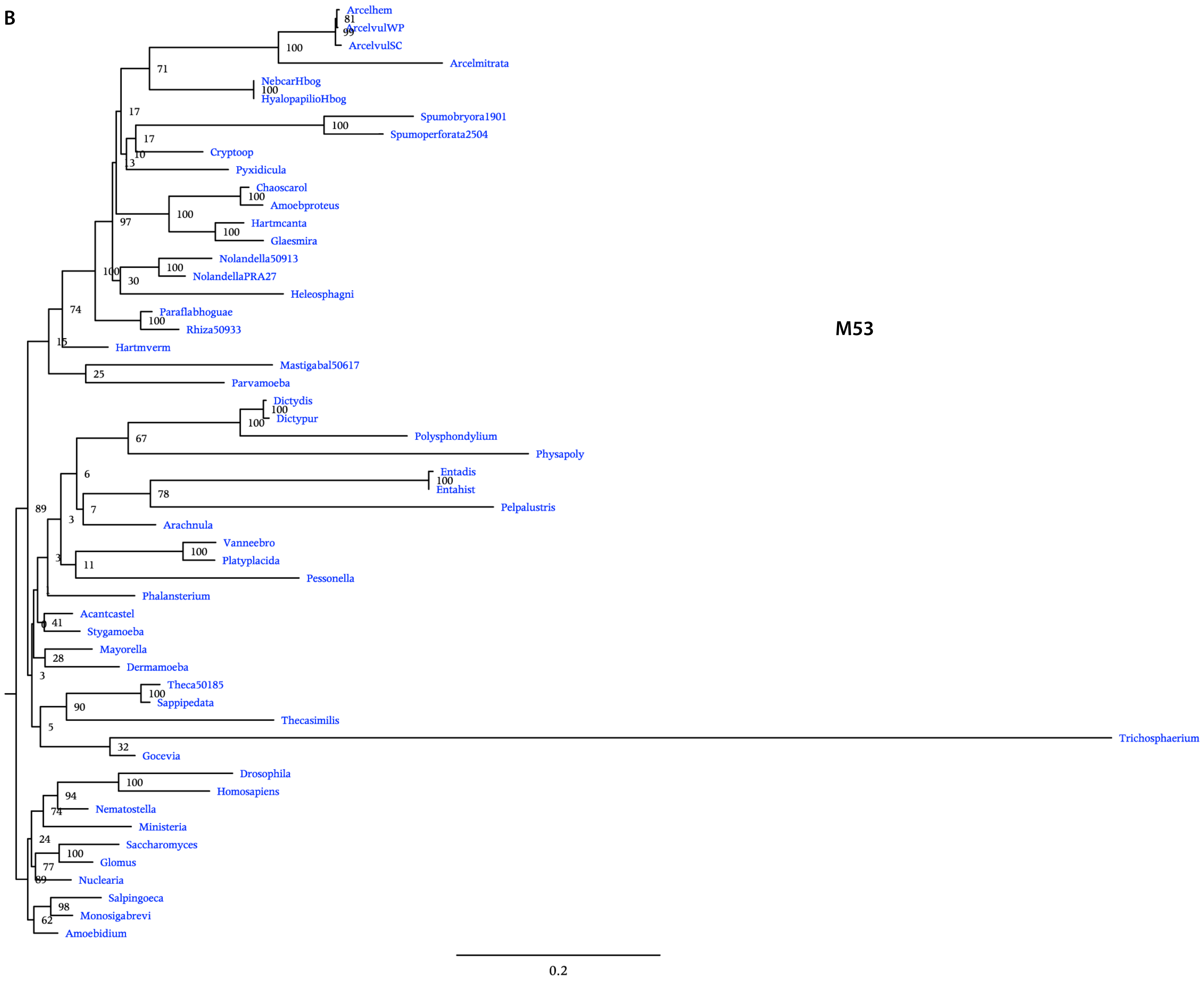
B
M53
Arcelhem
ArcelvulWP
ArcelvulSC
Arcelmitrata
NebcarHbog
HyalopapilioHbog
Spumobryora1901
Spumoperforata2504
Cryptoop
Pyxidicula
Chaoscarol
Amoebproteus
Hartmcanta
Glaesmira
Nolandella50913
NolandellaPRA27
Heleosphagni
Paraflabhoguae
Rhiza50933
Hartmverm
Mastigabal50617
Parvamoeba
Dictydis
Dictypur
Polysphondylium
Physapoly
Entadis
Entahist
Pelpalustris
Arachnula
Vanneebro
Platyplacida
Pessonella
Phalansterium
Acantcastel
Stygamoeba
Mayorella
Dermamoeba
Theca50185
Sappipedata
Thecasimilis
Trichosphaerium
Gocevia
Drosophila
Homosapiens
Nematostella
Ministeria
Saccharomyces
Glomus
Nuclearia
Salpingoeca
Monosigabrevi
Amoebidium
0.2

**C**

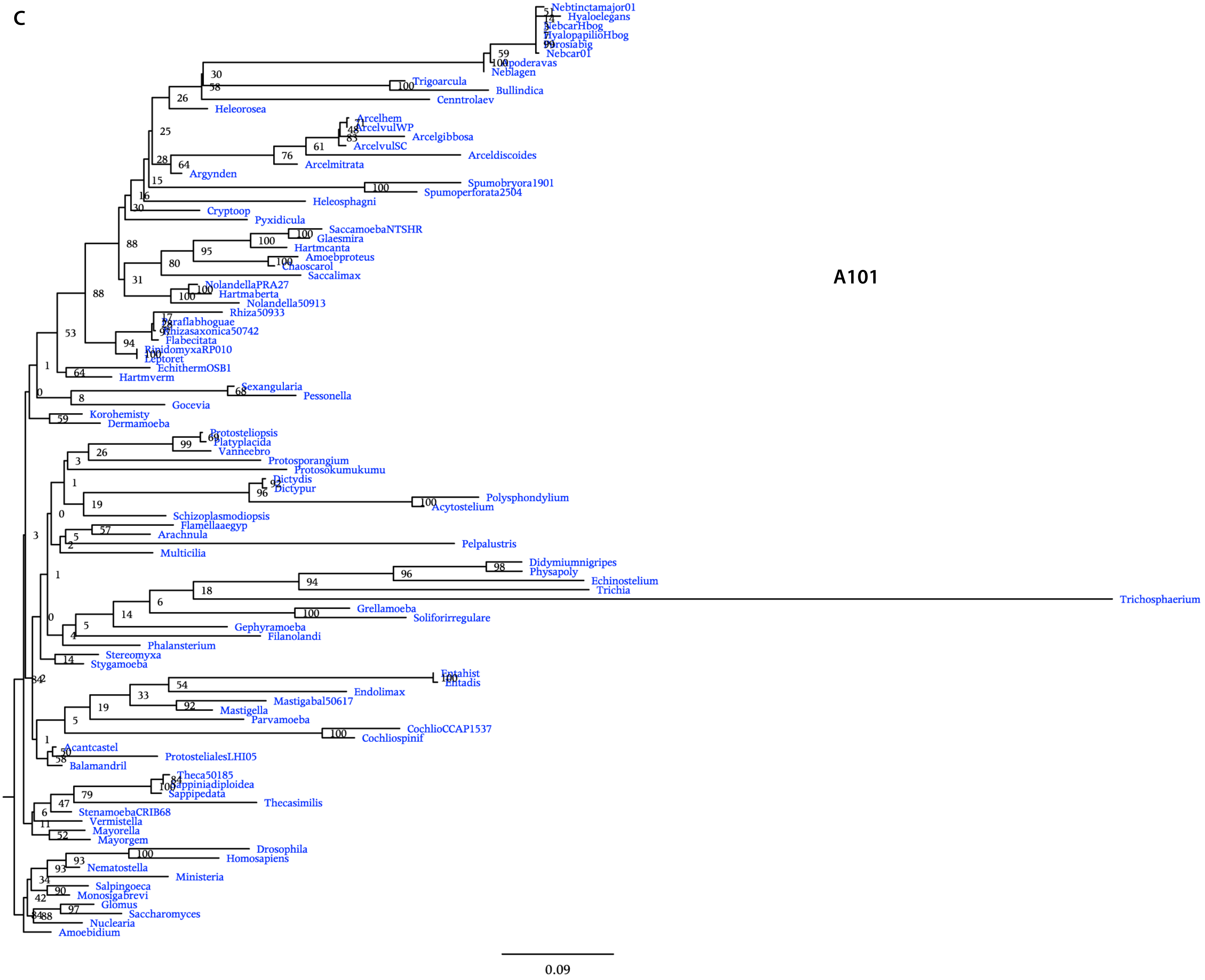
A101
Nebtinctamajor01
Hyaloelegans
NebcarHbog
HyalopapilioHbog
Nebcar01
Neblagen
Trigoarcula
Bullindica
Cenntrolaev
Heleorosea
Arcelhem
ArcelvulWP
Arcelgibbosa
ArcelvulSC
Arceldiscoides
Arcelmitrata
Argynden
Spumobryora1901
Spumoperforata2504
Heleosphagni
Cryptoop
Pyxidicula
SaccamoebaNTSHR
Glaesmira
Hartmcanta
Amoebproteus
Chaoscarol
Saccalimax
NolandellaPRA27
Hartmaberta
Nolandella50913
Rhiza50933
Paraflabhoguae
Rhizasaxonica50742
Flabecitata
RipidomyxaRP010
Leptoret
EchithermOSB1
Hartmverm
Sexangularia
Pessonella
Gocevia
Korohemisty
Dermamoeba
Protosteliopsis
Platyplacida
Vanneebro
Protosporangium
Protosokumukumu
Dictydis
Dictypur
Polysphondylium
Acytostelium
Schizoplasmodiopsis
Flamellaaegyp
Arachnula
Pelpalustris
Multicilia
Didymiumnigripes
Physapoly
Echinostelium
Trichia
Trichosphaerium
Grellamoeba
Soliforirregulare
Gephyramoeba
Filanolandi
Phalansterium
Stereomyxa
Stygamoeba
Entahist
Entadis
Endolimax
Mastigabal50617
Mastigella
Parvamoeba
CochlioCCAP1537
Cochliospinif
Acantcastel
ProtosteliialesLHI05
Balamandril
Theca50185
Sappiniadiploidea
Sappipedata
Thecasimilis
StenamoebaCRIB68
Vermistella
Mayorella
Mayorgem
Drosophila
Homosapiens
Nematostella
Ministeria
Salpingoeca
Monosigabrevi
Glomus
Saccharomyces
Nuclearia
Amoebidium
0.09

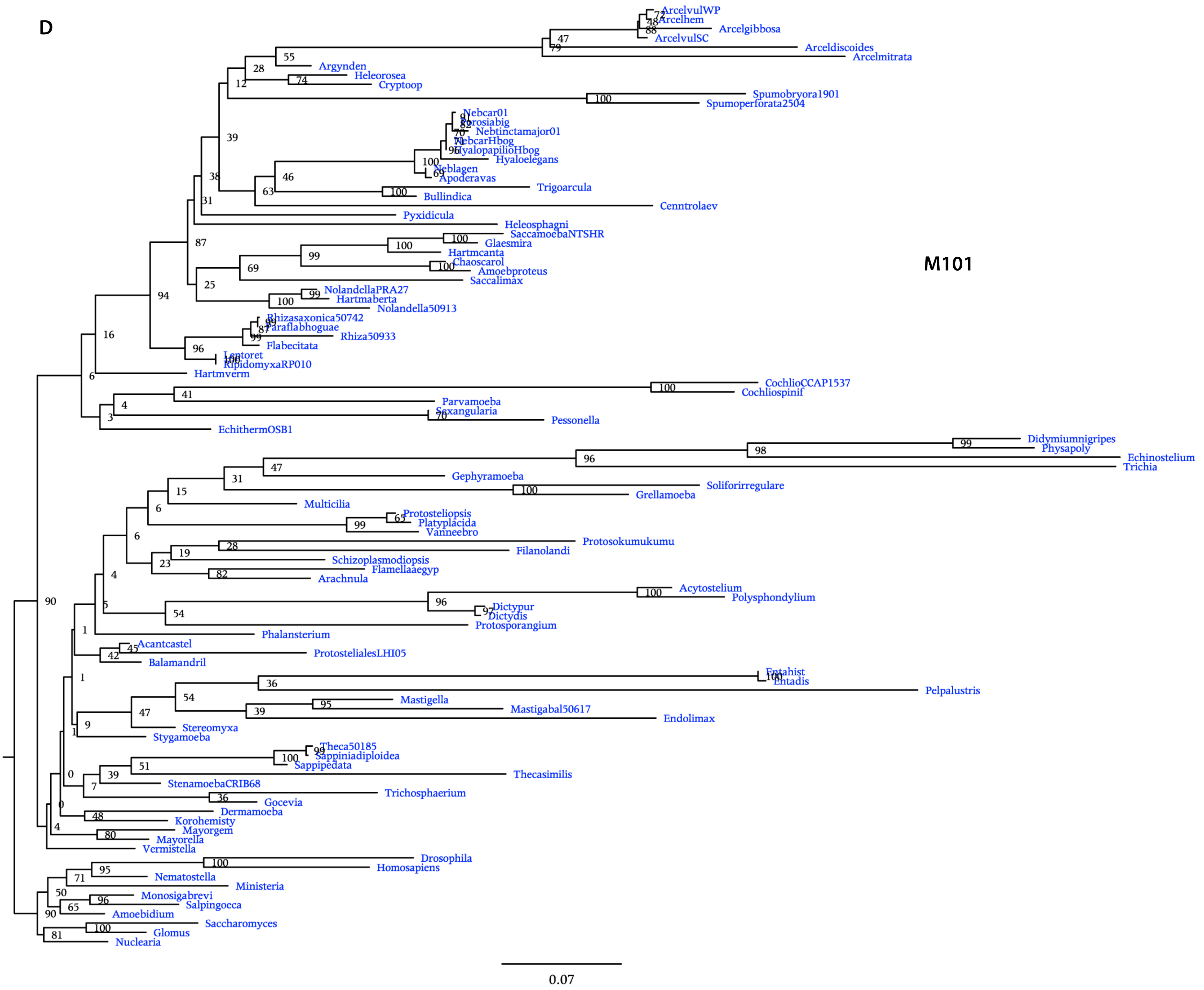
D
M101
ArcelvulWP
Arcelhem
Arcelgibbosa
ArcelvulSC
Arceldiscoides
Arcelmitrata
Argynden
Heleorosea
Cryptoop
Spumobryora1901
Spumoperforata2504
Nebcar01
Porosiabig
Nebtinctamajor01
NebcarHbog
HyalopapilioHbog
Hyaloelegans
Neblagen
Apoderavas
Trigoarcula
Bullindica
Cenntrolaev
Pyxidicula
Heleosphagni
SaccamoebaNTSHR
Glaesmira
Hartmcanta
Chaoscarol
Amoebproteus
Saccalimax
NolandellaPRA27
Hartmaberta
Nolandella50913
Rhizasaxonica50742
Paraflabhoguae
Rhiza50933
Flabecitata
Leptoret
RipidomyxaRP010
Hartmverm
CochlioCCAP1537
Cochliospinif
Parvamoeba
Sexangularia
Pessonella
EchithermOSB1
Didymiumnigripes
Physapoly
Echinostelium
Trichia
Gephyramoeba
Soliforirregulare
Grellamoeba
Multicilia
Protosteliopsis
Platyplacida
Vanneebro
Protosokumukumu
Filanolandi
Schizoplasmodiopsis
Flamellaaegyp
Arachnula
Acytostelium
Polysphondylium
Dictypur
Dictydis
Protosporangium
Phalansterium
Acantcastel
ProtostelialesLHI05
Balamandril
Entahist
Entadis
Pelpalustris
Mastigella
Mastigabal50617
Endolimax
Stereomyxa
Stygamoeba
Theca50185
Sappiniadiploidea
Sappipedata
Thecasimilis
StenamoebaCRIB68
Trichosphaerium
Gocevia
Dermamoeba
Korohemisty
Mayorgem
Mayorella
Vermistella
Drosophila
Homosapiens
Nematostella
Ministeria
Monosigabrevi
Salpingoeca
Amoebidium
Saccharomyces
Glomus
Nuclearia
0.07

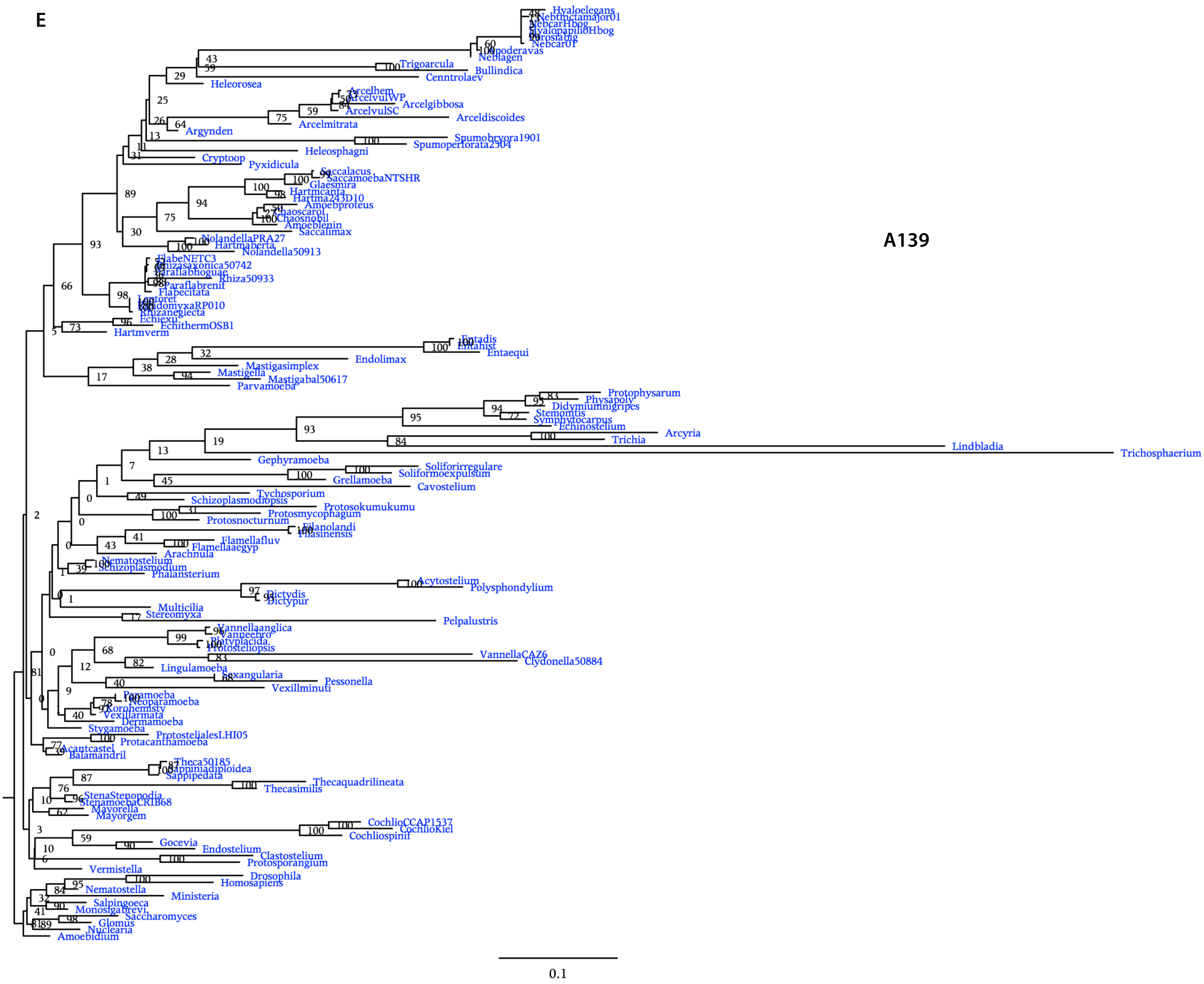
E
A139
Hyaloelegans
Nebtinctamajor01
NebcarHbog
HyalopapilioHbog
Porosatig
Nebcar01
Apoderavas
Neblagen
Bullindica
Trigoarcula
Cenntrolaev
Heleorosea
Arcelhem
ArcelvulWP
Arcelgibbosa
ArcelvulSC
Arceldiscoides
Arcelmitrata
Argynden
Spumobryora1901
Spumoperforata2504
Heleosphagni
Cryptoop
Pyxidicula
Saccalacus
SaccamoebaNTSHR
Glaesmira
Hartmcanta
Hartma243D10
Amoebproteus
Chaoscarol
Chaosnobil
Amoeblenin
Saccalimax
NolandellaPRA27
Hartmaberta
Nolandella50913
FlabeNETC3
Rhizasaxonica50742
Paraflabhoguae
Rhiza50933
Paraflabrenif
Flabecitata
Leptoret
RhidomyxaRP010
Rhizaneglecta
Echiexu
EchithermOSB1
Hartmverm
Entadis
Entahist
Entaequi
Endolimax
Mastigasimplex
Mastigella
Mastigabal50617
Parvamoeba
Protophysarum
Physapoly
Didymiumnigripes
Stemonitis
Symphytocarpus
Echinostelium
Arcyria
Trichia
Lindbladia
Trichosphaerium
Gephyramoeba
Soliforirregulare
Soliformoexpulsum
Grellamoeba
Cavostelium
Tychosporium
Schizoplasmodiopsis
Protosokumukumu
Protosmycophagum
Protosnocturnum
Filanolandi
Filasinensis
Flamellafluv
Flamellaaegyp
Arachnula
Nematostelium
Schizoplasmodium
Phalansterium
Acytostelium
Polysphondylium
Dictydis
Dictypur
Multicilia
Stereomyxa
Pelpalustris
Vannellaanglica
Vanneebro
Platyplacida
Protosteliopsis
VannellaCAZ6
Clydonella50884
Lingulamoeba
Sexangularia
Pessonella
Vexillminuti
Paramoeba
Neoparamoeba
Korotnevella
Vexillarmata
Dermamoeba
Stygamoeba
ProtostelialesLHI05
Protacanthamoeba
Acantcastel
Balamandril
Theca50185
Sappiniadiploidea
Sappipedata
Thecaquadrilineata
Thecasimilis
StenaStenopodia
StenamoebaCRIB68
Mayorella
Mayorgem
CochlioCCAP1537
CochlioKiel
Cochliospinif
Gocevia
Endostelium
Clastostelium
Protosporangium
Vermistella
Drosophila
Homosapiens
Nematostella
Ministeria
Salpingoeca
Monosigabrevi
Saccharomyces
Glomus
Nuclearia
Amoebidium
0.1

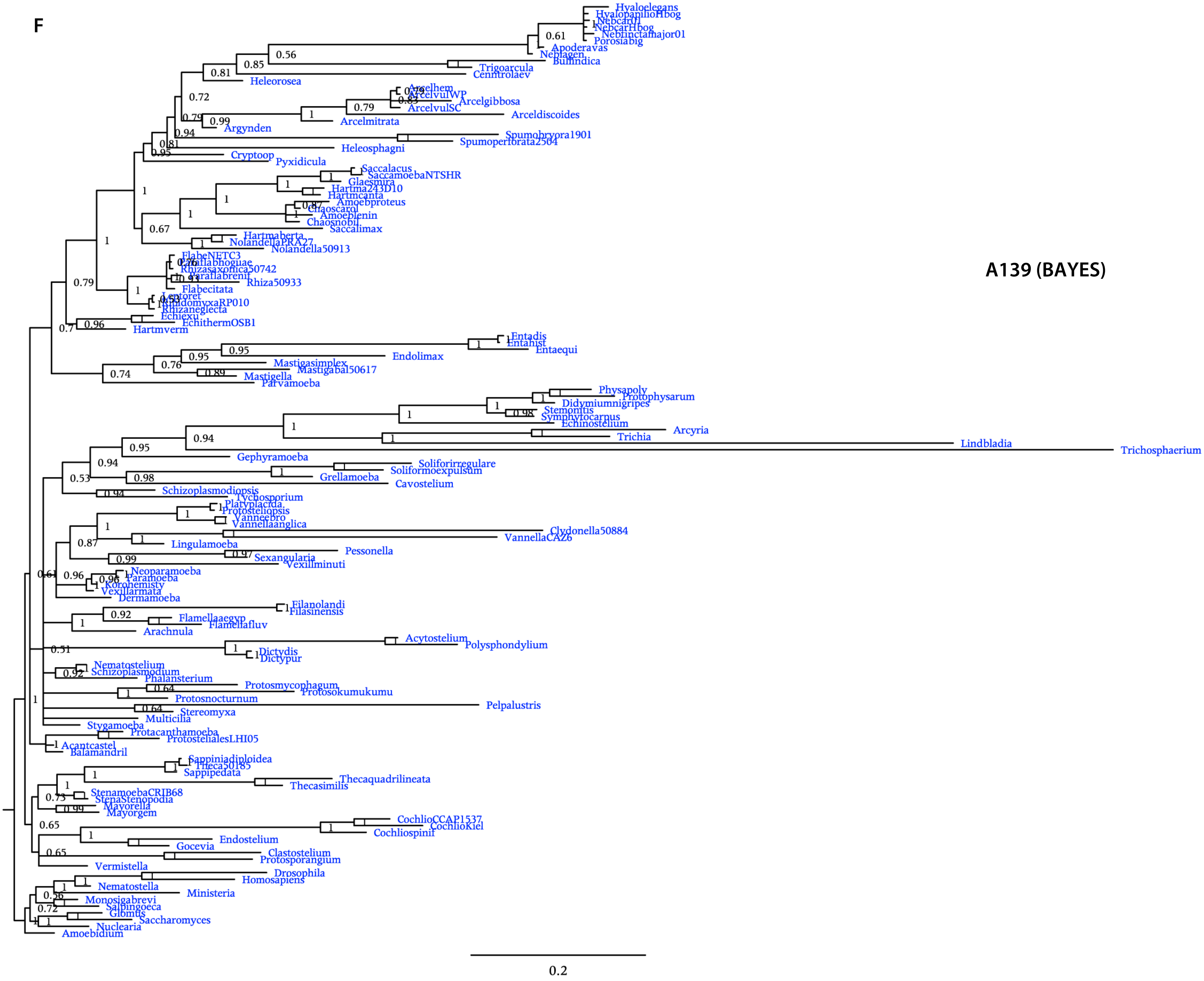
F
A139 (BAYES)
Hyaloelegans
HyalopapilioHbog
Nebcar01
NebcarHbog
Nebtinctamajor01
Porosiabig
Apoderavas
Neblagen
Bullinindica
Trigoarcula
Centrolaev
Heleorosea
Arcelhem
ArcelvulWP
Arcelgibbosa
ArcelvulSC
Arceldiscoides
Arcelmitrata
Argynden
Spumobryora1901
Spumoperforata2504
Heleosphagni
Cryptoop
Pyxidicula
Saccalacus
SaccamoebaNTSHR
Glaesmira
Hartma243D10
Hartmcanta
Amoebproteus
Chaoscarol
Amoeblenin
Chaosnobil
Saccalimax
Hartmaberta
NolandellaPRA27
Nolandella50913
FlabeNETC3
Paraflabhoguae
Rhizasaxonica50742
Paraflabrenif
Rhiza50933
Flabecitata
Leptoret
RhizomyxaRP010
Rhizaneglecta
Echiexu
EchithermOSB1
Hartmverm
Entadis
Entahist
Entaequi
Endolimax
Mastigasimplex
Mastigabal50617
Mastigella
Parvamoeba
Physapoly
Protophysarum
Didymiumnigripes
Stemonitis
Symphytocarpus
Echinostelium
Arcyria
Trichia
Lindbladia
Trichosphaerium
Gephyramoeba
Soliforirregulare
Soliformoexpulsum
Grellamoeba
Cavostelium
Schizoplasmodiopsis
Tychosporium
Platyplacida
Protosteliopsis
Vanneebro
Vannellaanglica
Clydonella50884
VannellaCAZ6
Lingulamoeba
Pessonella
Sexangularia
Vexilliminuti
Neoparamoeba
Paramoeba
Korotnevella
Korohemisty
Vexillarmata
Dermamoeba
Filanolandi
Filasinensis
Flamellaaegyp
Flamellafluv
Arachnula
Acytostelium
Polysphondylium
Dictydis
Dictypur
Nematostelium
Schizoplasmodium
Phalansterium
Protosmycophagum
Protosokumukumu
Protosnocturnum
Pelpalustris
Stereomyxa
Multicilia
Stygamoeba
Protacanthamoeba
ProtostelialesLHI05
Acantcastel
Balamandril
Sappiniadiploidea
Theca50185
Sappipedata
Thecaquadrilineata
Thecasimilis
StenamoebaCRIB68
StenaStenopodia
Mayorella
Mayorgem
CochlioCCAP1537
CochlioKiel
Cochliospinif
Endostelium
Gocevia
Clastostelium
Protosporangium
Vermistella
Drosophila
Homosapiens
Nematostella
Ministeria
Monosigabrevi
Salpingoeca
Glomus
Saccharomyces
Nuclearia
Amoebidium
0.2

G

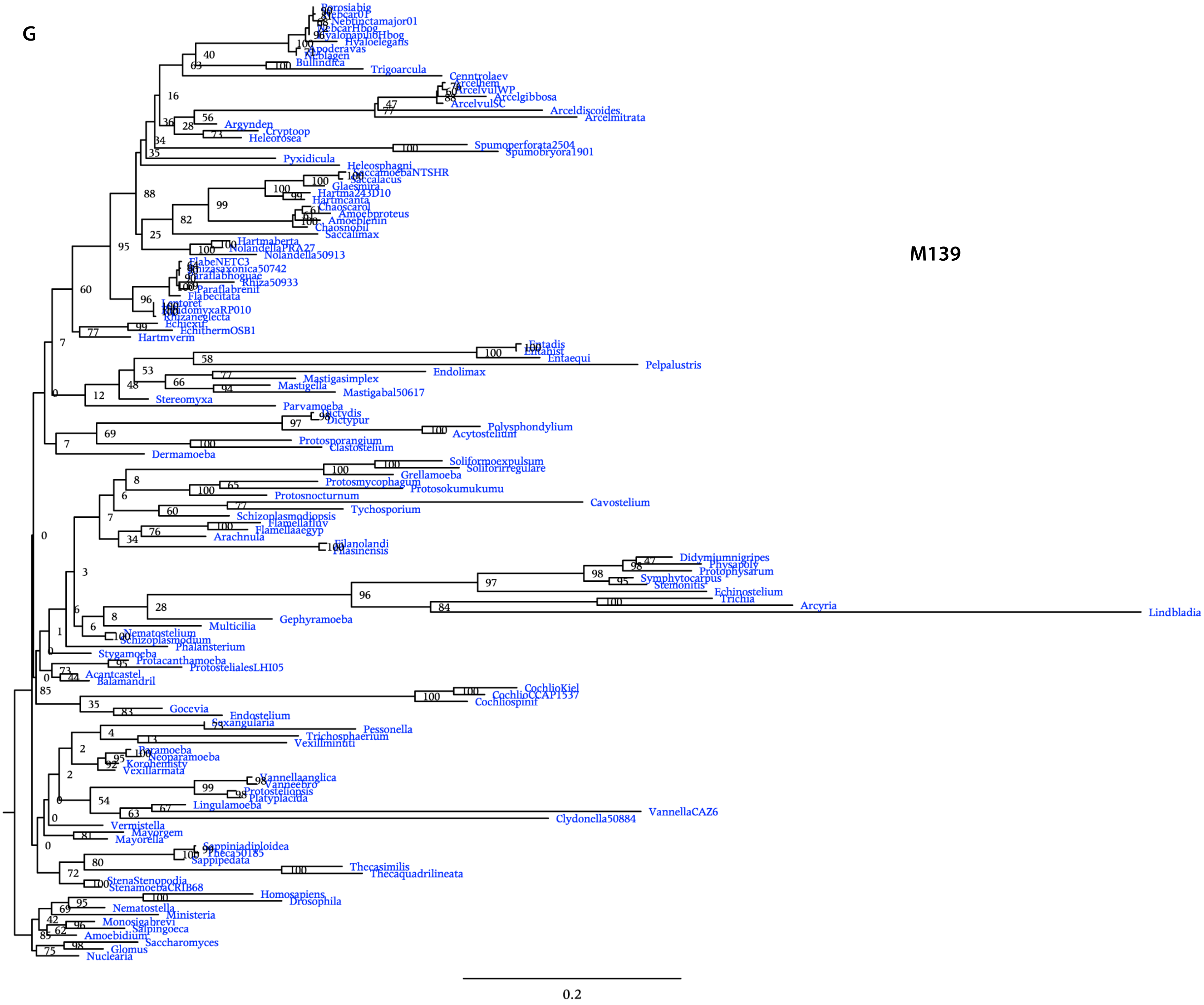

M139
Porosiabig
Nebcar0P
Nebtinctamajor01
NebcarHbog
HyalopapilioHbog
Hyaloelegans
Apoderavas
Neblagen
Bullinidea
Trigoarcula
Cenntrolaev
Arcelhem
ArcelvulWP
Arcelgibbosa
ArcelvulSC
Arceldiscoides
Arcelmitrata
Argynden
Cryptoop
Heleorosea
Spumoperforata2504
Spumobryora1901
Pyxidicula
Heleosphagni
SaccamoebaNTSHR
Saccalacus
Glaesmira
Hartma243D10
Hartmcanta
Chaoscarol
Amoebproteus
Amoeblenin
Chaosnobil
Saccalimax
Hartmaberta
NolandellaPRA27
Nolandella50913
FlabeNETC3
Rhizasaxonica50742
Paraflabhoguae
Rhiza50933
Paraflabrenif
Flabecitata
Leptoret
RhizidomyxaRP010
Rhizaneglecta
Echiexil
EchithermOSB1
Hartmverm
Entadis
Entahist
Entaequi
Pelpalustris
Endolimax
Mastigasimplex
Mastigella
Mastigabal50617
Stereomyxa
Parvamoeba
Dictydis
Dictypur
Polysphondylium
Acytostelium
Protosporangium
Clastostelium
Dermamoeba
Soliformoexpulsum
Soliformirregulare
Grellamoeba
Protosmycophagum
Protosokumukumu
Protosnocturnum
Cavostelium
Tychosporium
Schizoplasmodiopsis
Flamellafluv
Flamellaaegyp
Arachnula
Filanolandi
Filasinensis
Didymiumnigripes
Physapoly
Protophysarum
Symphytocarpus
Stemonitis
Echinostelium
Trichia
Arcyria
Lindbladia
Multicilia
Gephyramoeba
Nematostelium
Schizoplasmodium
Phalansterium
Stygamoeba
Protacanthamoeba
ProtostelialesLHI05
Acantcastel
Balamandril
CochlioKiel
CochlioCCAP1537
Cochliospinif
Gocevia
Endostelium
Saxangularia
Pessonella
Trichosphaerium
Vexillminuti
Paramoeba
Neoparamoeba
Korohemisty
Vexillarmata
Vannellaanglica
Vanneebro
Protosteliopsis
Platyplacida
Lingulamoeba
VannellaCAZ6
Clydonella50884
Vermistella
Mayorgem
Mayorella
Sappiniadiploidea
Theca50185
Sappinpedata
Thecasimilis
Thecaquadrilineata
StenaStenopodia
StenamoebaCRIB68
Homosapiens
Drosophila
Nematostella
Ministeria
Monosigabrevi
Salpingoeca
Amoebidium
Saccharomyces
Glomus
Nuclearia
0.2

H

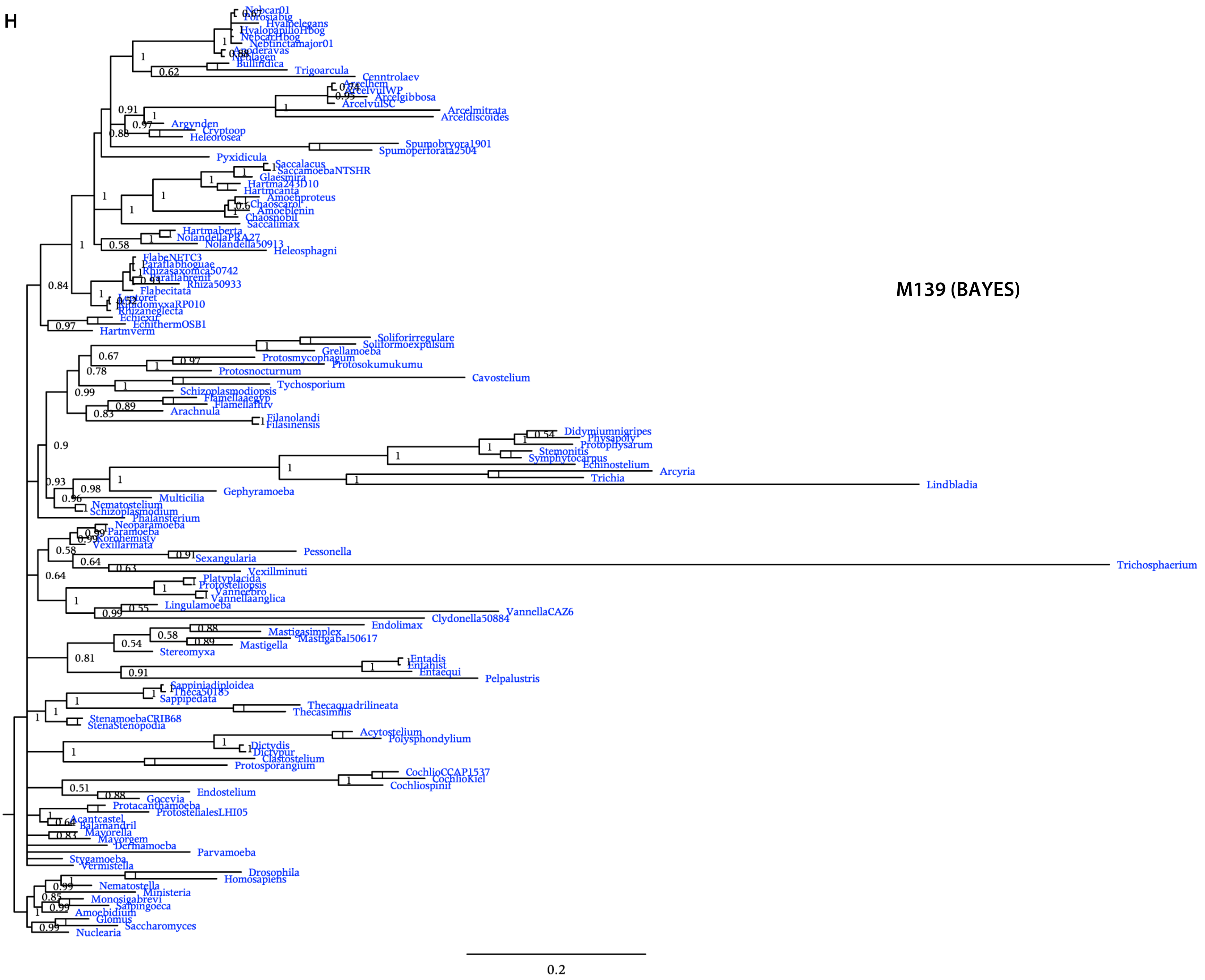

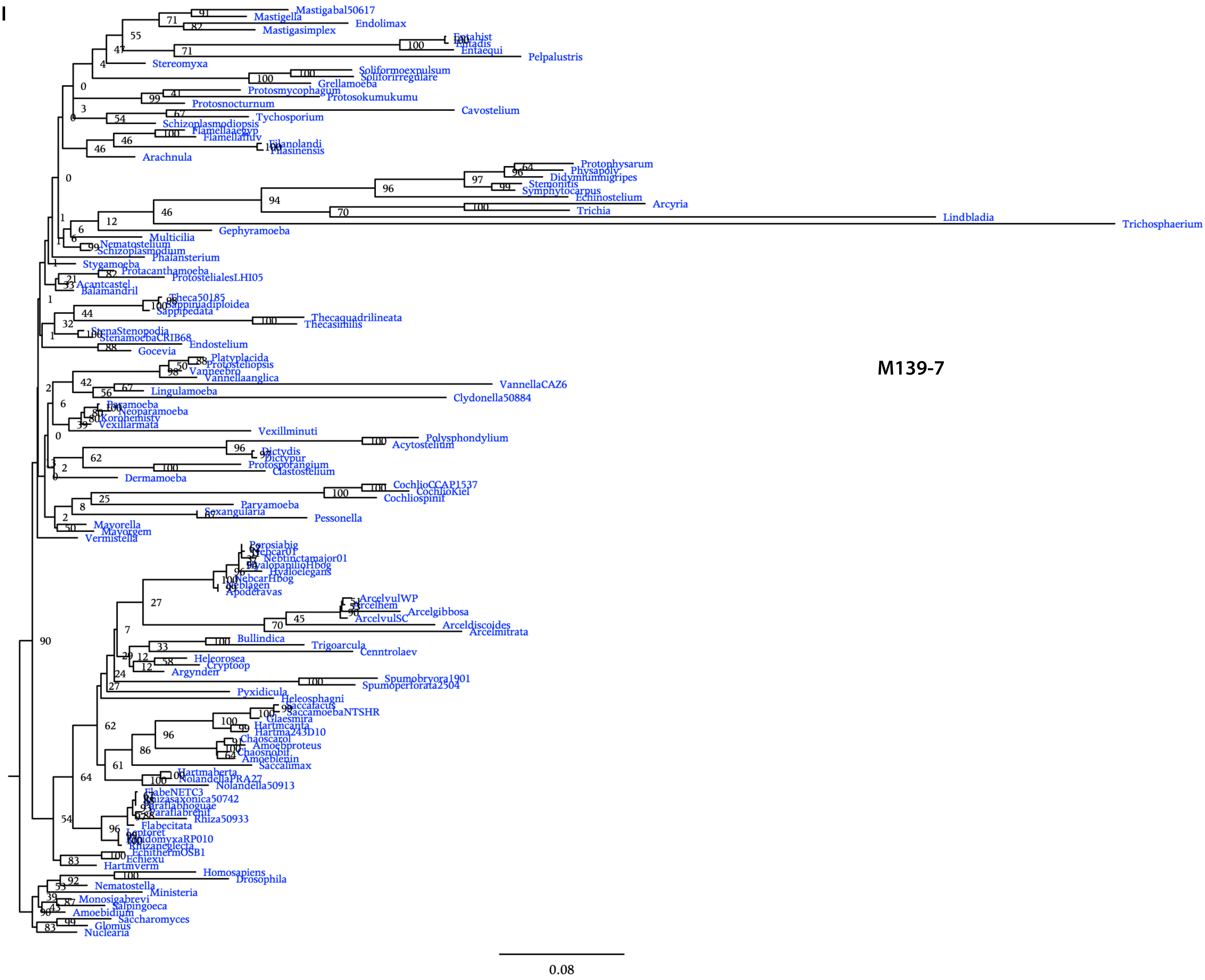
I
M139-7
Mastigabal50617
Mastigella
Endolimax
Mastigasimplex
Entahist
Entdis
Entaequi
Pelpalustris
Stereomyxa
Soliformoexpulsum
Soliforirregulare
Grellamoeba
Protosmycophagum
Protosokumukumu
Protosnocturnum
Cavostelium
Tychosporium
Schizoplasmodiopsis
Flamellaaegyp
Flamellafluv
Filanolandi
Filasinensis
Arachnula
Protophysarum
Physapoly
Didymiumnigripes
Stemonitis
Symphytocarpus
Echinostelium
Arcyria
Trichia
Lindbladia
Trichosphaerium
Gephyramoeba
Multicilia
Nematostelium
Schizoplasmodium
Phalansterium
Stygamoeba
Protacanthamoeba
ProtostelialesLHI05
Acantcastel
Balamandril
Theca50185
Sappiniadiploidea
Sappipedata
Thecaquadrilineata
Thecasimilis
StenaStenopodia
StenamoebaCRIB68
Endostelium
Gocevia
Platyplacida
Protosteliopsis
Vanneebro
Vannellaanglica
VannellaCAZ6
Lingulamoeba
Clydonella50884
Paramoeba
Neoparamoeba
Korotnevella
Vexillarmata
Vexillminuti
Polysphondylium
Acytostelium
Dictydis
Dictypur
Protosporangium
Clastostelium
Dermamoeba
CochlioCCAP1537
CochlioKiel
Cochliospinif
Paryamoeba
Sexangularia
Pessonella
Mayorella
Mayorgem
Vermistella
Porosiabig
Nebcar01
Nebtinctamajor01
HyalopapilioHbog
Hyaloelegans
NebcarHbog
Nebcollaris
Apodera vas
ArcelvulWP
Arcelhem
Arcelgibbosa
ArcelvulSC
Arceldiscoides
Arcelmitrata
Bullindica
Trigonopyxis
Centropyxis
Heleorosea
Cryptodifflugia
Argynnia
Spumobryora1901
Spumoperforata2504
Pyxidicula
Heleosphagni
Saccamoeba
SaccamoebaNTSHR
Glaesmira
Hartmcanta
Hartma243D10
Chaoscarol
Amoebproteus
Chaosnobil
Amoeblenin
Saccalimax
Hartmaberta
NolandellaPRA27
Nolandella50913
FlabeNETC3
Rhizasaxonica50742
Paraflabhoguae
Paraflabrenit
Rhiza50933
Flabecitata
Leptoret
LeptomyxaRP010
Rhizaneglecta
EchithermOSB1
Echiexu
Hartmverm
Homosapiens
Drosophila
Nematostella
Ministeria
Monosigabrevi
Salpingoeca
Amoebidium
Saccharomyces
Glomus
Nuclearia
0.08

J

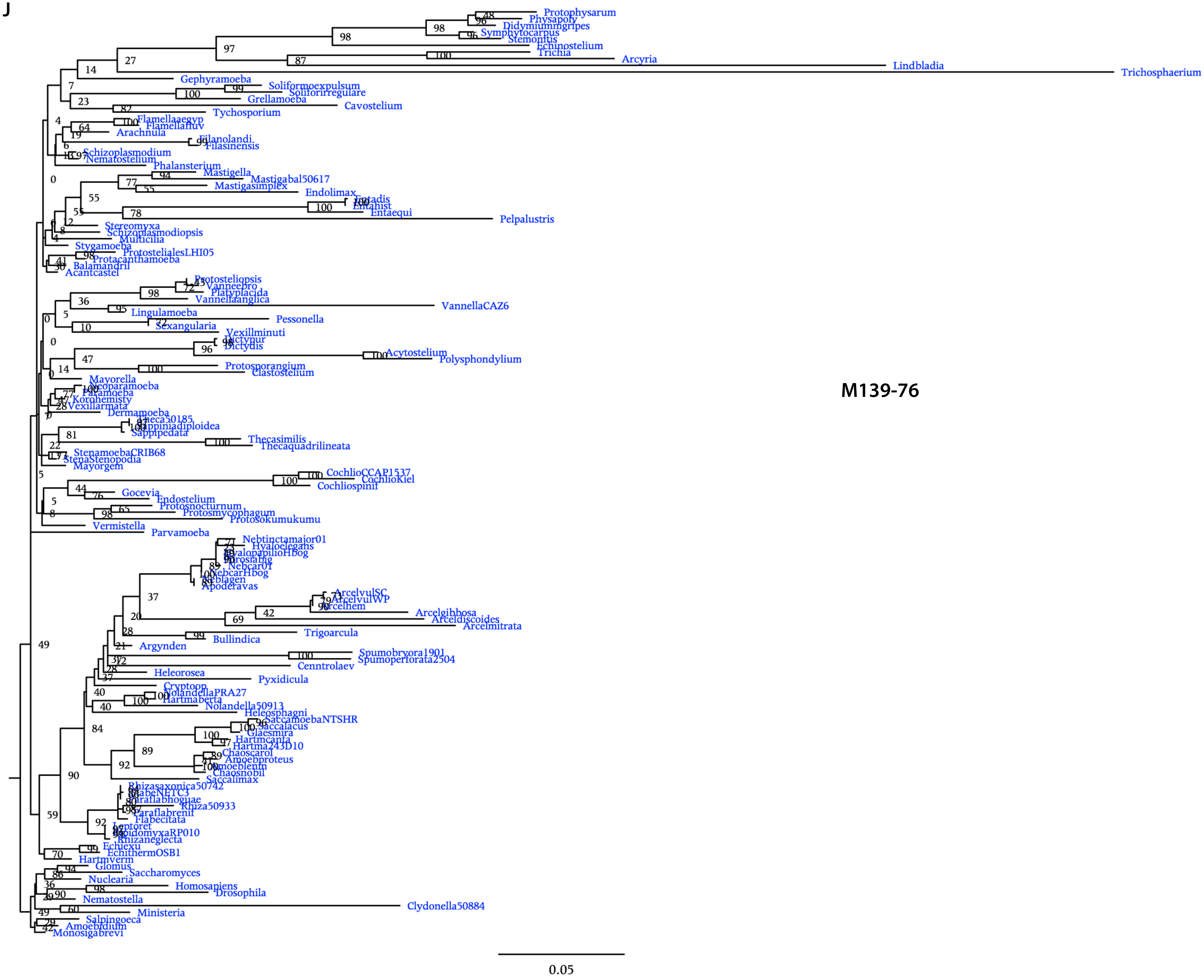
Protophysarum
Physapoly
Didymiumnigripes
Symphytocarpus
Stemonitis
Echinostelium
Trichia
Arcyria
Lindbladia
Trichosphaerium
Gephyramoeba
Soliformoexpulsum
Soliforirregulare
Grellamoeba
Cavostelium
Tychosporium
Flamellaaegyp
Flamellafluv
Arachnula
Filanolandi
Filasinensis
Schizoplasmodium
Nematostelium
Phalansterium
Mastigella
Mastigabal50617
Mastigasimplex
Endolimax
Entadis
Entahist
Entaequi
Pelpalustris
Stereomyxa
Schizoplasmodiopsis
Multicilia
Stygamoeba
ProtostelialesLHI05
Protacanthamoeba
Balamandril
Acantcastel
Protosteliopsis
Vanneebro
Platyplacida
Vannellaanglica
VannellaCAZ6
Lingulamoeba
Pessonella
Sexangularia
Vexillminuti
Dictypur
Dictydis
Acytostelium
Polysphondylium
Protosporangium
Clastostelium
Mayorella
Neoparamoeba
Paramoeba
Korotnevella
Vexillarmata
Dermamoeba
Theca50185
Sappiniadiploidea
Sappipedata
Thecasimilis
Thecaquadrilineata
StenamoebaCRIB68
StenaStenopodia
Mayorgem
CochlioCCAP1537
CochlioKiel
Cochliospinif
Gocevia
Endostelium
Protosnocturnum
Protosmycophagum
Protosokumukumu
Vermistella
Parvamoeba
Nebtinctamajor01
Hyaloelegans
HyalopapilioHbog
Nebcar01
NebcarHbog
Apoderavas
ArcelvulSC
ArcelvulWP
Arcelhem
Arcelgibbosa
Arceldiscoides
Arcelmitrata
Trigoarcula
Bullindica
Argynden
Spumobryora1901
Spumoperforata2504
Cenntrolaev
Heleorosea
Pyxidicula
NolandellaPRA27
Hartmaberta
Nolandella50913
Heleosphagni
SaccamoebaNTSHR
Saccalacus
Glaesmira
Hartmcanta
Hartma243D10
Chaoscarol
Amoebproteus
Amoeblenin
Chaosnobil
Saccalimax
Rhizasaxonica50742
Rhiza50933
Paraflabrenif
Flabecitata
Leptoret
Rhizaneglecta
Echiexu
EchithermOSB1
Hartmverm
Glomus
Saccharomyces
Nuclearia
Homosapiens
Drosophila
Nematostella
Clydonella50884
Ministeria
Salpingoeca
Amoebidium
Monosigabrevi
M139-76
0.05

K

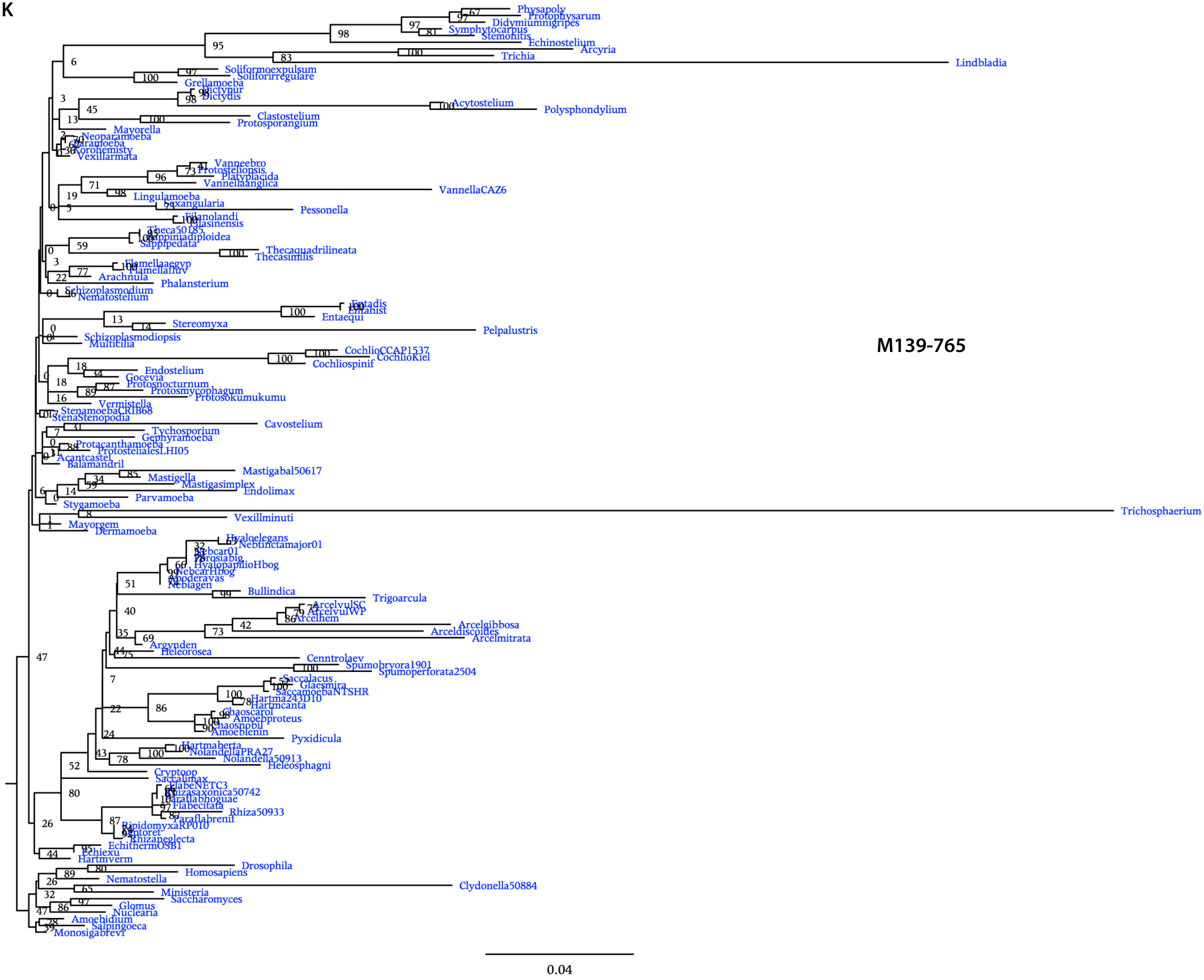

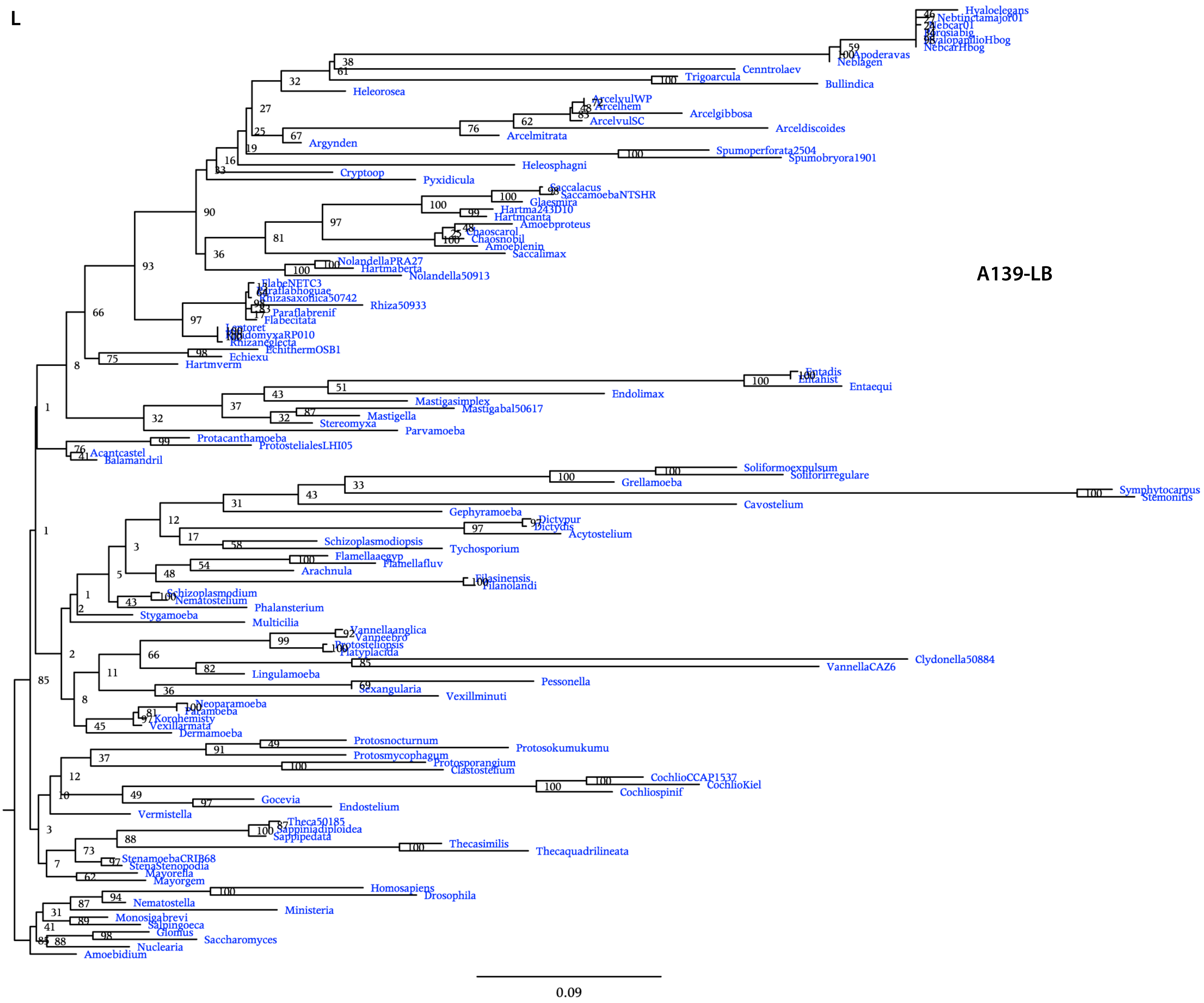
L
A139-LB
0.09

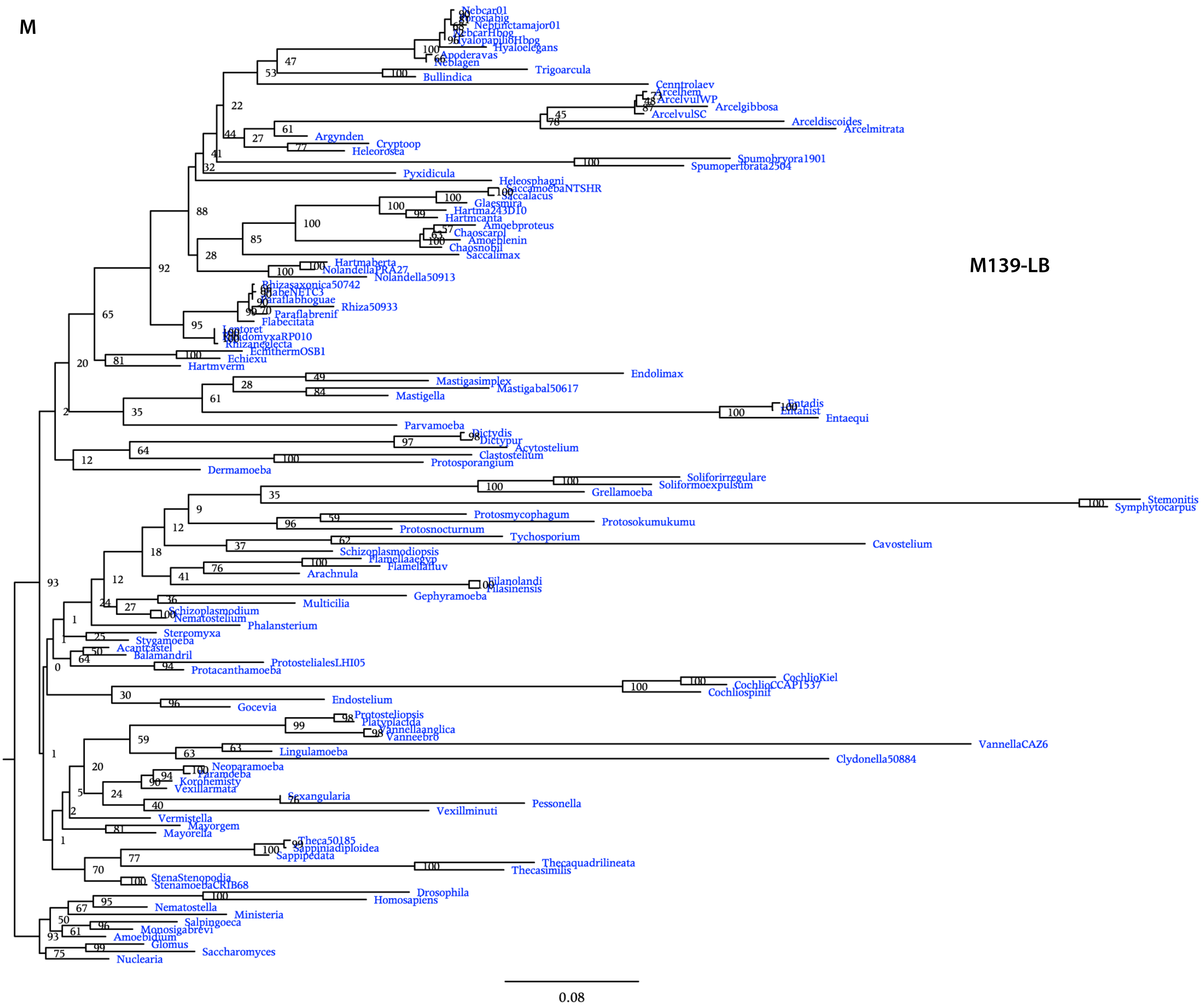
M
M139-LB
Nebcar01
Nebtinctamajor01
NebcarHbog
HyalopapilioHbog
Hyaloelegans
Apoderavas
Neblagen
Trigoarcula
Bullindica
Cenntrolaev
Arcelhem
ArcelvulWP
Arcelgibbosa
ArcelvulSC
Arceldiscoides
Arcelmitrata
Argynden
Cryptoop
Heleorosea
Spumobryora1901
Spumoperforata2504
Pyxidicula
Heleosphagni
SaccamoebaNTSHR
Saccalacus
Glaesmira
Hartma243D10
Hartmcanta
Amoebproteus
Chaoscarol
Amoeblenin
Chaosnobil
Saccalimax
Hartmaberta
NolandellaPRA27
Nolandella50913
Rhizasaxonica50742
Paraflabhoguae
Rhiza50933
Paraflabrenif
Flabecitata
Rhizaneglecta
EchithermOSB1
Echiexu
Hartmverm
Endolimax
Mastigasimplex
Mastigabal50617
Mastigella
Entadis
Entaqui
Parvamoeba
Dictydis
Dictypur
Acytostelium
Clastostelium
Protosporangium
Dermamoeba
Soliforirregulare
Soliformoexpulsum
Grellamoeba
Stemonitis
Symphytocarpus
Protosmycophagum
Protosokumukumu
Protosnocturnum
Tychosporium
Cavostelium
Schizoplasmodiopsis
Flamellaaegyp
Flamellafluv
Arachnula
Filanolandi
Filasinensis
Gephyramoeba
Multicilia
Schizoplasmodium
Nematostelium
Phalansterium
Stereomyxa
Stygamoeba
Acantcastel
Balamandril
ProtostelialesLHI05
Protacanthamoeba
CochlioKiel
CochlioCCAP1537
Cochliospinif
Endostelium
Gocevia
Protosteliopsis
Platyplacida
Vannellaanglica
Vanneebro
VannellaCAZ6
Lingulamoeba
Clydonella50884
Neoparamoeba
Paramoeba
Korotnevella
Vexillarmata
Sexangularia
Pessonella
Vexillminuti
Vermistella
Mayorgem
Mayorella
Theca50185
Sappiniadiploidea
Sappipedata
Thecaquadrilineata
Thecasimilis
StenaStenopodia
StenamoebaCRIB68
Drosophila
Homosapiens
Nematostella
Ministeria
Salpingoeca
Monosigabrevi
Amoebidium
Glomus
Saccharomyces
Nuclearia
0.08

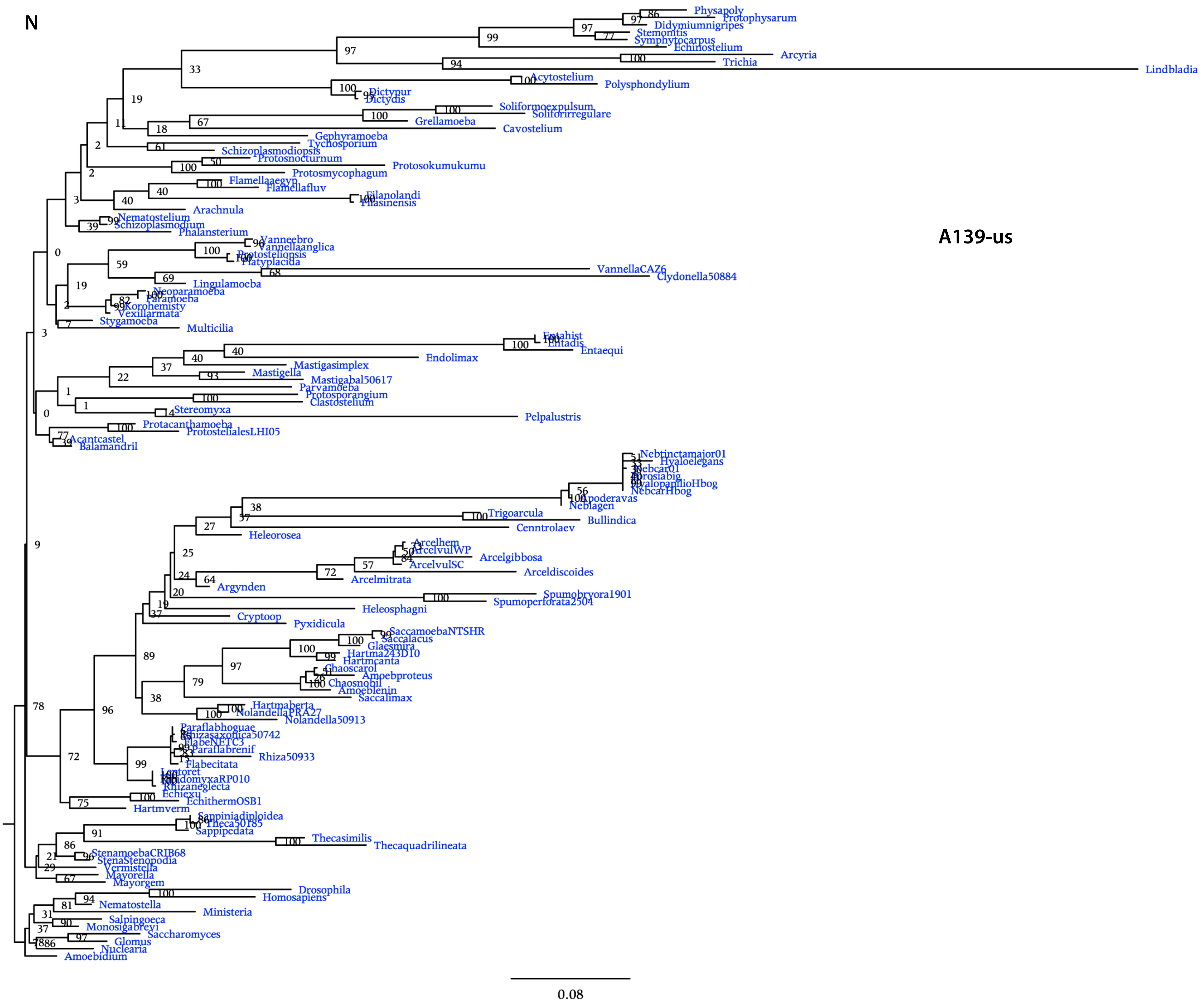
N
A139-us
Physapoly
Protophysarum
Didymiumnigripes
Stemonitis
Symphytocarpus
Echinostelium
Arcyria
Trichia
Lindbladia
Acytostelium
Polysphondylium
Dictyur
Dictydis
Soliformoexpulsum
Soliforirregulare
Grellamoeba
Cavostelium
Gephyramoeba
Tychosporium
Schizoplasmodiopsis
Protosnocturnum
Protosokumukumu
Protosmycophagum
Flamellaaegyp
Flamellafluv
Filanolandi
Filasinensis
Arachnula
Nematostelium
Schizoplasmodium
Phalansterium
Vanneebro
Vannellaanglica
Protosteliopsis
Platyplacida
VannellaCAZ6
Clydonella50884
Lingulamoeba
Neoparamoeba
Paramoeba
Korohemisty
Vexillarmata
Stygamoeba
Multicilia
Entahist
Entadis
Entaequi
Endolimax
Mastigasimplex
Mastigella
Mastigabal50617
Parvamoeba
Protosporangium
Clastostelium
Stereomyxa
Pelpalustris
Protacanthamoeba
ProtostelialesLHI05
Acantcastel
Balamandril
Nebtinctamajor01
Hyaloelegans
Nebcar01
Porosiabig
HyalopapilioHbog
NebcarHbog
Apoderavas
Neblagen
Bullindica
Trigoarcula
Cenntrolaev
Heleorosea
Arcelhem
ArcelvulWP
Arcelgibbosa
ArcelvulSC
Arceldiscoides
Arcelmitrata
Argynden
Spumobryora1901
Spumoperforata2504
Heleosphagni
Cryptoop
Pyxidicula
SaccamoebaNTSHR
Saccalacus
Glaesmira
Hartma243D10
Hartmcanta
Chaoscarol
Amoebproteus
Chaosnobil
Amoeblenin
Saccalimax
Hartmaberta
NolandellaPRA27
Nolandella50913
Paraflabhoguae
Rhizasaxonica50742
FlabeNETC3
Paraflabrenif
Rhiza50933
Flabecitata
Leptoret
RhizomyxaRP010
Rhizaneglecta
Echiexu
EchithermOSB1
Hartmverm
Sappiniadiploidea
Theca50185
Sappipedata
Thecasimilis
Thecaquadrilineata
StenamoebaCRIB68
StenaStenopodia
Vermistella
Mayorella
Mayorgem
Drosophila
Homosapiens
Nematostella
Ministeria
Salpingoeca
Monosigabrevi
Saccharomyces
Glomus
Nuclearia
Amoebidium
0.08

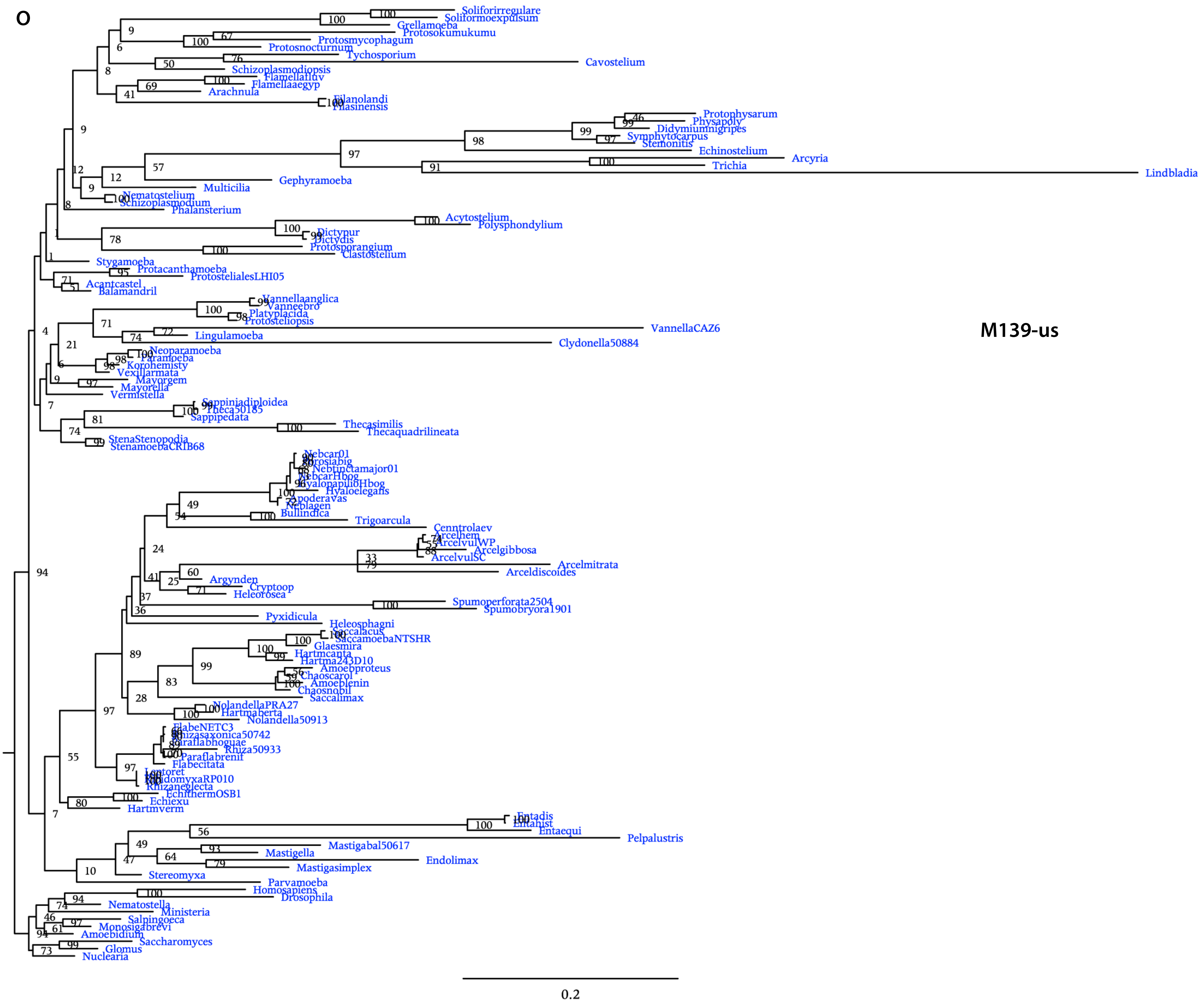
O
M139-us
0.2

P

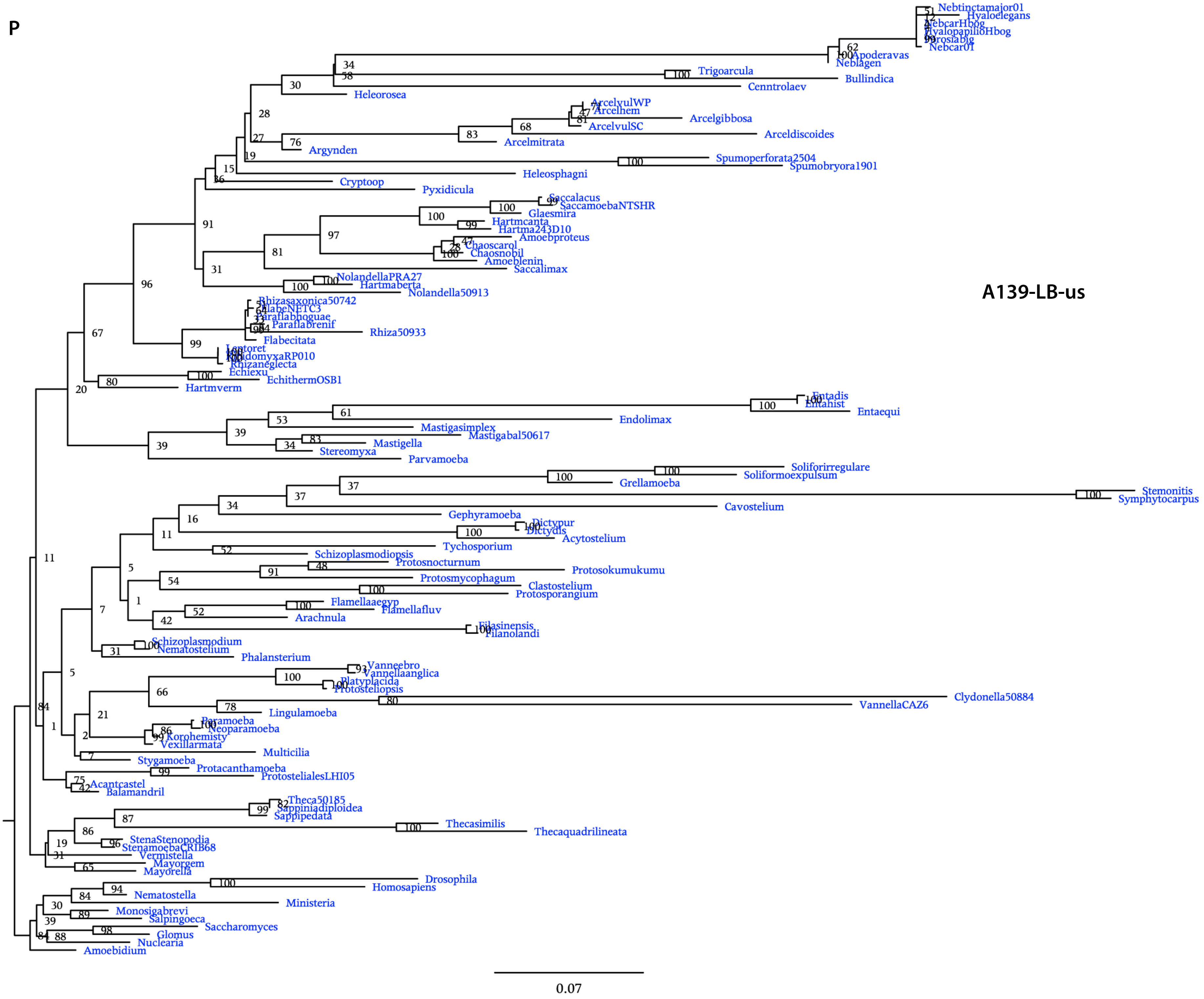

Nebtinctamajor01
Hyaloelegans
NebcarHbog
HyalopapilioHbog
Porosiabig
Apoderavas
Nebcar01
Neblagen
Trigoarcula
Bullindica
Cenntrolaev
Heleorosea
ArcelvulWP
Arcelhem
Arcelgibbosa
ArcelvulSC
Arceldiscoides
Arcelmitrata
Argynden
Spumoperforata2504
Spumobryora1901
Heleosphagni
Cryptoop
Pyxidicula
Saccalacus
SaccamoebaNTSHR
Glaesmira
Hartmcanta
Hartma243D10
Amoebproteus
Chaoscarol
Chaosnobil
Amoeblenin
Saccalimax
NolandellaPRA27
Hartmaberta
Nolandella50913
Rhizasaxonica50742
FlabeNETC3
Paraflabhoguae
Paraflabrenif
Rhiza50933
Flabecitata
Leptoret
Rhizaneglecta
Echiexu
EchithermOSB1
Hartmverm
Entadis
Entahist
Entaequi
Endolimax
Mastigasimplex
Mastigabal50617
Mastigella
Stereomyxa
Parvamoeba
Soliforirregulare
Soliformoexpulsum
Grellamoeba
Stemonitis
Symphytocarpus
Cavostelium
Gephyramoeba
Dictypur
Dictydis
Acytostelium
Tychosporium
Schizoplasmodiopsis
Protosnocturnum
Protosokumukumu
Protosmycophagum
Clastostelium
Protosporangium
Flamellaaegyp
Flamellafluv
Arachnula
Filasinensis
Filanolandi
Schizoplasmodium
Nematostelium
Phalansterium
Vanneebro
Vannellaanglica
Platyplacida
Protosteliopsis
Clydonella50884
VannellaCAZ6
Lingulamoeba
Paramoeba
Neoparamoeba
Korohemisty
Vexillarmata
Multicilia
Stygamoeba
Protacanthamoeba
ProtostelialesLHI05
Acantcastel
Balamandril
Theca50185
Sappiniadiploidea
Sappipedata
Thecasimilis
Thecaquadrilineata
StenaStenopodia
StenamoebaCRIB68
Vermistella
Mayorgem
Mayorella
Drosophila
Homosapiens
Nematostella
Ministeria
Monosigabrevi
Salpingoeca
Saccharomyces
Glomus
Nuclearia
Amoebidium
A139-LB-us
0.07

Q

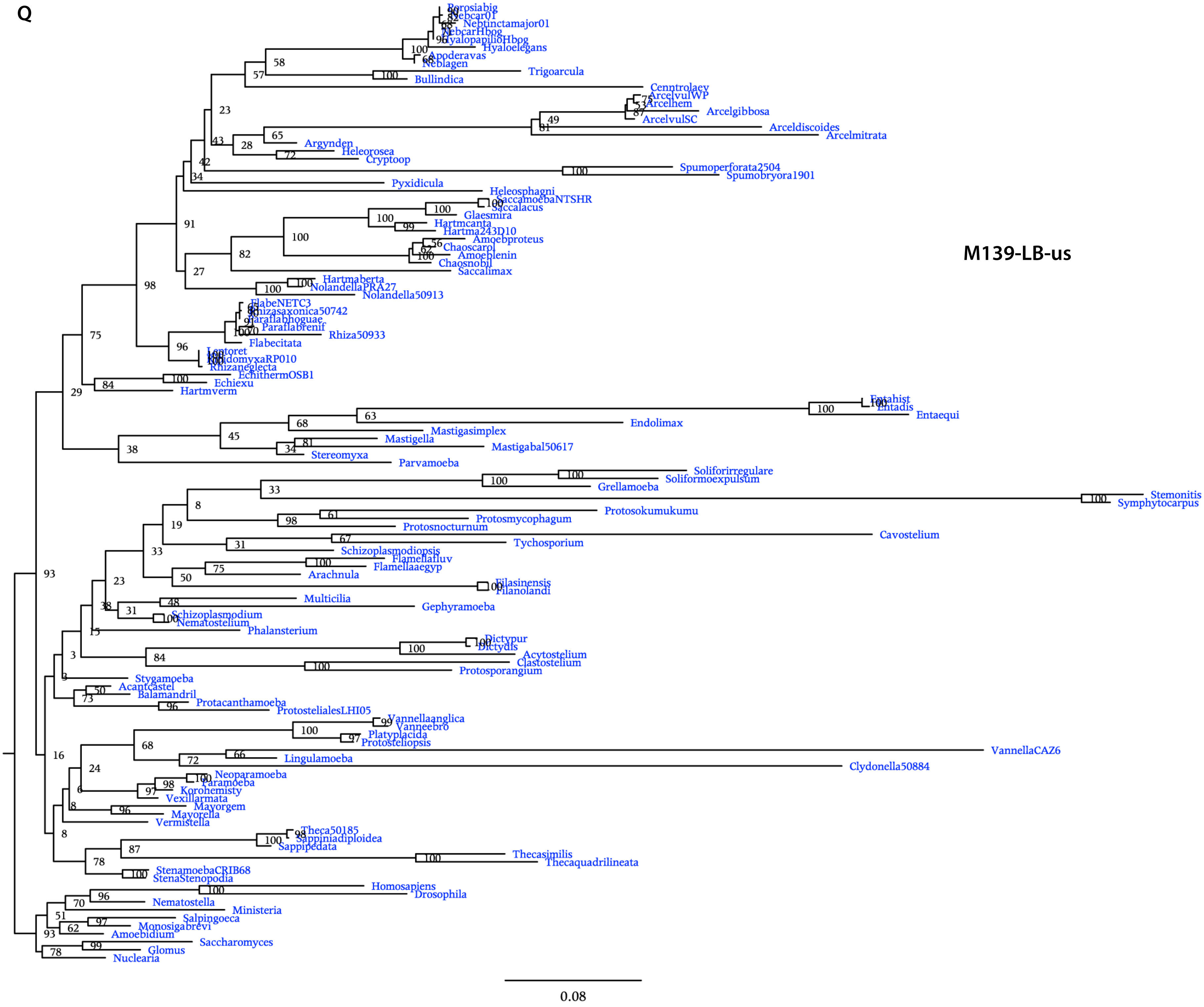
M139-LB-us
Nebcar01
Nebtinctamajor01
NebcarHbog
HyalopapilioHbog
Hyaloelegans
Apoderavas
Neblagen
Trigoarcula
Bullindica
Cenntrolaev
ArcelvulWP
Arcelhem
Arcelgibbosa
ArcelvulSC
Arceldiscoides
Arcelmitrata
Argynden
Heleorosea
Cryptoop
Spumoperforata2504
Spumobryora1901
Pyxidicula
Heleosphagni
SaccamoebaNTSHR
Saccalacus
Glaesmira
Hartmcanta
Hartma243D10
Amoebproteus
Chaoscarol
Amoeblenin
Chaosnobil
Saccalimax
Hartmaberta
NolandellaPRA27
Nolandella50913
FlabeNETC3
Schizasaxonica50742
Paraflabhoguae
Paraflabrenif
Rhiza50933
Flabecitata
Leptoret
Rhizaneglecta
EchithermOSB1
Echiexu
Hartmverm
Entahist
Entadis
Entaequi
Endolimax
Mastigasimplex
Mastigella
Mastigabal50617
Stereomyxa
Parvamoeba
Soliforirregulare
Soliformoexpulsum
Grellamoeba
Stemonitis
Symphytocarpus
Protosokumukumu
Protosmycophagum
Protosnocturnum
Cavostelium
Tychosporium
Schizoplasmodiopsis
Flamellafluv
Flamellaaegyp
Arachnula
Filasinensis
Filanolandi
Multicilia
Gephyramoeba
Schizoplasmodium
Nematostelium
Phalansterium
Dictypur
Dictydis
Acytostelium
Clastostelium
Protosporangium
Stygamoeba
Acantcastel
Balamandril
Protacanthamoeba
ProtostelialesLHI05
Vannellaanglica
Vanneebro
Platyplacida
Protosteliopsis
VannellaCAZ6
Lingulamoeba
Clydonella50884
Neoparamoeba
Paramoeba
Korohemisty
Vexillarmata
Mayorgem
Mayorella
Vermistella
Theca50185
Sappiniadiploidea
Sappipedata
Thecasimilis
Thecaquadrilineata
StenamoebaCRIB68
StenaStenopodia
Homosapiens
Drosophila
Nematostella
Ministeria
Salpingoeca
Monosigabrevi
Amoebidium
Saccharomyces
Glomus
Nuclearia
0.08

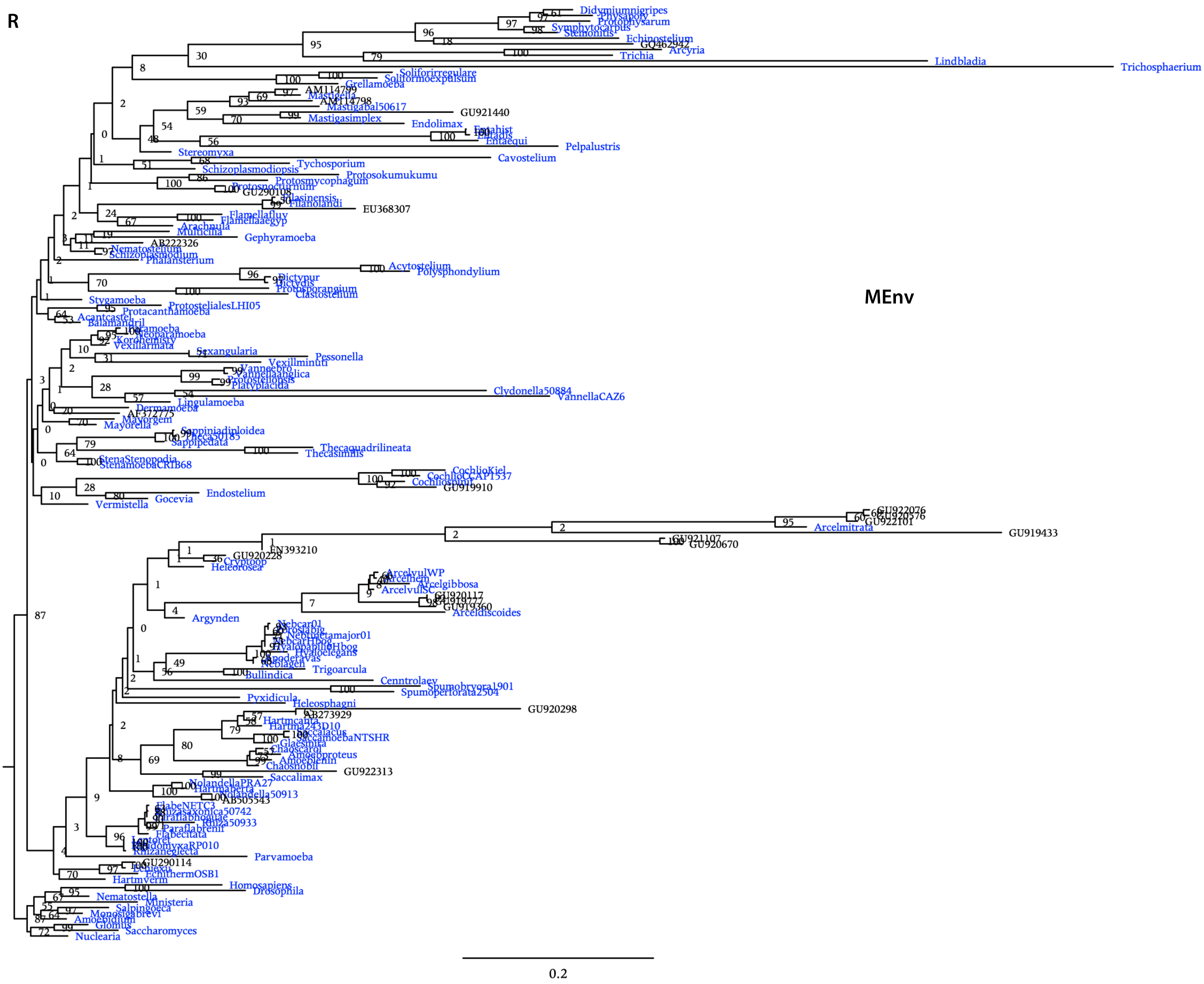
R
MEnv
0.2
