# Supplementary material for: Comprehensive Phylogenetic Reconstruction of Amoebozoa Based on Concatenated Analyses of SSU-rDNA and Actin Genes
Source: PLoS One. 2011 Jul 28;6(7):e22780. doi: 10.1371/journal.pone.0022780 (PMC3145751; doi:10.1371/journal.pone.0022780)
Supplement: Text S1 — (DOC) [file pone.0022780.s006.doc]

**Supplementary Text S1**

Comparative analyses of phylogenetic reconstructions

*Comparative analyses of Boostrap Supports*

In the multiple analyses performed, as the number of taxa increases, the support for morphologically defined groups tends to increase. This tendency is clear both in reconstructions with automated or manual removal of ambiguous sites (eg. Mastigamoebidae and Pelobiontidae, Table 3). However, higher-level relationships present a less clear pattern, as only a few of the proposed groups are supported, and the majority is not recovered at all (Table 3). Removing the fastest evolving rate class tends to break apart moderately supported groups (e.g. Vannellida and Acanthamoebida), but retain the highly supported ones (e.g. Amoebidae, Dictyostellida). However, further removal of the second and third fastest evolving rate classes results in significant disruption of clades. Removing either the long-branched or the unstable taxa does not have a large effect on bootstrap support for the remaining taxa (Table 3). However, removing both usually increases support for moderately supported groups, and leaves highly supported nodes unchanged. The addition of environmental sequences seems to have little effect on bootstrap supports, except for the eventual reduction on poorly supported groups (e.g. Arcellinida, Table 3).

*Comparative analyses of Treeness indices*

The treeness index is a simple measure of signal to noise ratio in phylogenetic reconstructions, and in our analyses tend to increase as more taxa are added (Table 5). This pattern is seen for both the automatic and manual removal of ambiguous sites, although the automatic reconstructions tend to have slightly higher treeness indices (e.g. M139 scores 0.35 while the comparable A139 scores 0.40, Table 5). Removing long branched taxa from the dataset has the direct effect of increasing the treeness index (e.g. M139-LB scores 0.41 compared to 0.35 in M139, Table 5). However, we do not believe this translates to a better reconstruction, but is in fact an expected outcome of how the index is calculated, i.e., reconstructions without the long-branched taxa do not inherently have more signal, they merely have a more optimal signal to noise ratio (Table 5). Removal of unstable taxa does not affect the treeness index, and removal of both unstable and long branched taxa results in an increased treeness index, which is attributable to the same increase when only long-branched taxa are removed (Table 5). The treeness index decreases as the fastest evolving rate classes are removed, indicating that these sites probably contain useful information for reconstruction. Further, the addition of environmental sequences promotes only a slight increase in the signal to noise ratio.

*Comparative analysis of Average Leaf Stabilities*

The average leaf stability is perhaps the most useful index for assessing performance in reconstructing groups of interest (Table 5, Supplementary Table S2). There is a decreasing tendency in average stability as more taxa are added for datasets with automated removal of ambiguous sites, with the largest dataset having a significantly lower overall stability (A139=0.8±0.01) than the smaller dataset (A53=0.84±0.02). The trend is not present in datasets with manually removed ambiguous sites, where stabilities are not significantly different as more taxa are added (Table 5). However, manual datasets are significantly more stable than their automated counterparts (e.g. M101=0.86±0.01 versus A101=0.82±0.02, Table 5). Removal of either long-branched taxa or unstable taxa does not have a significant impact in overall stability. However, the joint removal of both significantly increases stability (M139-LB-us=0.88±0.01 versus M139=0.84±0.01). Removal of the fastest evolving rate class does not impact stability, nevertheless, further removal of the second and third fastest evolving rate classes leads to a large drop (M139-765=0.73±0.01), indicating again that important information is contained in those sites. The dataset with manually removed ambiguous sites, and deleted long-branched + unstable taxa displays the optimal combination of measures, however does not enable assessment of all groups of interest. Yet, it is useful as a comparison point in confirming moderately supported groups in other reconstructions.
